# Distinct postnatal trajectories of mouse dendritic epidermal T cells and Langerhans cells independent of microbiota

**DOI:** 10.1126/sciadv.aec9180

**Published:** 2026-08-14

**Authors:** David Obwegs, Alexander Oschwald, Lara M. Koetter, Cylia Crisand, Sidney Doerr, Kerstin Bruder, Solveig Runge, Nisreen Ghanem, Vidmante Fuchs, Marleen Eckert, Christian Koengeter, Anna-Maria Schaffer, Lukas Amann, Sophia Papaioannou, Tim Rollenske, Julia Kolter, Daniel Erny, Marco Prinz, Susana Minguet, Wolfgang W. Schamel, Philipp Henneke, Stephan P. Rosshart, Katrin Kierdorf, Sagar

**Affiliations:** ^1^ Department of Medicine II, Faculty of Medicine, Medical Center–University of Freiburg, 79106 Freiburg, Germany.; ^2^ Faculty of Biology, University of Freiburg, 79104 Freiburg, Germany.; ^3^ Institute of Neuropathology, Faculty of Medicine, Medical Center–University of Freiburg, 79106 Freiburg, Germany.; ^4^ Institute for Infection Prevention and Control, Faculty of Medicine, Medical Center–University of Freiburg, 79106 Freiburg, Germany.; ^5^ Department of Prosthetic Dentistry, University Hospital, LMU Munich, 80336 Munich, Germany.; ^6^ Department of Microbiome Research, University Hospital Erlangen, Friedrich-Alexander-Universität (FAU), 91054 Erlangen, Germany.; ^7^ Department of Medicine 1, University Hospital Erlangen, Friedrich-Alexander-Universität (FAU), 91054 Erlangen, Germany.; ^8^ Department of Synthetic Immunology, Faculty of Biology, University of Freiburg, 79104 Freiburg, Germany.; ^9^ Centre for Chronic Immunodeficiency (CCI), Faculty of Medicine, University of Freiburg, 79106 Freiburg, Germany.; ^10^ Institute of Molecular Medicine and Experimental Immunology, University of Bonn, University Hospital Bonn, 53127 Bonn, Germany.; ^11^ Center for Brain Research and Advancements in Neuroimmunology (BRAIN), Faculty of Medicine, University of Freiburg, 79106 Freiburg, Germany.; ^12^ Signalling Research Centres BIOSS and CIBSS, University of Freiburg, 79104 Freiburg, Germany.; ^13^ Department of Immunology, Faculty of Biology, University of Freiburg, 79104 Freiburg, Germany.

## Abstract

The mouse epidermis harbors two key resident immune populations—dendritic epidermal T cells (DETCs), a subset of invariant γδ T cells, and Langerhans cells (LCs), specialized tissue-resident macrophages—both of which play critical roles in immune surveillance, barrier integrity, and tissue homeostasis. While their fetal origin has been defined, the mechanisms governing their postnatal maturation remain poorly understood. Here, we present a combined immunophenotypic and single-cell transcriptomic map of DETC and LC development from late embryogenesis through adulthood in mice. We delineate distinct differentiation trajectories characterized by dynamic changes in morphology, proliferation, and transcriptional programming. Using γδ T cell deficient mice, we show that LC maturation proceeds independently of canonical γδDETCs, likely due to compensatory αβDETCs. Analysis of germfree mice and wildlings further demonstrates that the postnatal DETC and LC differentiation is independent of microbial colonization. Comparative analysis with developing human epidermis reveals partially conserved differentiation programs. Together, our findings define core principles underlying establishment of the epidermal immune niche.

## INTRODUCTION

The skin is one of the largest and most complex organs in vertebrates. It separates the organism from the environment. It provides protection against dehydration, pathogens, toxins, and ultraviolet radiation, while it enables sensory perception of stimuli such as pain, heat, and touch at the same time (*1*). Beyond its structural role in maintaining hydration and shielding internal tissues, the skin functions as a highly specialized immune organ. Anatomically, the skin is divided into two major layers—the epidermis and the dermis—both of which host diverse immune cell repertoires that contribute to tissue homeostasis, immune surveillance, and tolerance to commensal microbiota (*2*). The epidermis forms the outermost layer of the skin and consists of tightly packed, interconnected avascular layers of keratinocytes (*3*). Despite its compact structure, the mouse epidermis contains a distinct and relatively stable resident immune cell community composed of two key populations: dendritic epidermal T cells (DETCs), unique γδ T cells expressing an invariant Vγ5Vδ1 T cell receptor (TCR) lacking junctional diversity (according to the J. S. Heilig and S. Tonegawa nomenclature) (*4*, *5*), and Langerhans cells (LCs), a specialized subset of dendritic cell–like tissue-resident macrophages (*6*).

In steady state, DETCs are critical for epidermal integrity because their absence leads to increased keratinocyte apoptosis, likely due to reduced production of insulin-like growth factor 1 (*7*). Moreover, LCs mediate tolerance to commensal microbiota, preemptive immunity, and immune surveillance (*8*, *9*). Their absence in adulthood has been associated with disorganized keratinocyte layering, the emergence of autoreactive T cells, and compromised barrier function (*10*–*12*). The functions of DETCs and LCs have been extensively studied in mouse models of skin inflammation, wound healing, and cancer. DETCs contribute to wound repair by sensing stressed keratinocytes and producing cytokines and growth factors such as interferon-γ (IFN-γ), tumor necrosis factor–α (TNF-α), and keratinocyte growth factor (*13*–*16*). LCs, in turn, act as antigen-presenting cells during infection and inflammation and are critical for preventing autoimmunity and limiting inflammatory responses (*17*, *18*). Dysregulation of either cell type in the adult epidermis has been associated with impaired tissue repair and inflammatory skin diseases.

Both DETCs and LCs populate the mouse epidermis in the perinatal phase, entering the tissue niche in parallel or shortly after one another (*19*, *20*). Once established, they remain within this niche throughout life, maintaining their populations via endogenous proliferation in steady state (*21*, *22*). Bona fide adult LCs are thought to originate from erythromyeloid progenitors (EMPs) in the embryonic yolk sac. Between embryonic day 8.5 (E8.5) and E10.5, EMPs colonize the fetal liver and give rise to fetal monocytes, which later migrate to the dermis. Around birth, LC progenitors transmigrate into the developing epidermis (*20*, *23*). DETCs originate from fetal thymic precursors, where they are the first T cells to develop in fetal mouse thymus. These precursors may themselves arise from the yolk sac (*19*). Following epidermal entry, both DETCs and LCs undergo postnatal adaptation within the developing niche, acquiring their characteristic ramified morphology and functional properties (*20*, *24*). Prior studies have identified essential signals instructing LC differentiation, including transforming growth factor–β (TGF-β); its receptor, interleukin-34 (IL-34); and colony-stimulating factor 1 receptor (CSF1R) signaling (*25*–*27*). While these pathways are indispensable for the generation and maintenance of adult LCs, selection and upkeep of intraepithelial T cells 1 (Skint1) has been shown to be central for DETC development and differentiation in the mouse epidermis (*28*–*31*).

In the adult mouse skin, it was suggested that DETC and LCs contribute to coordinated spatial tiling and reciprocal signaling. DETCs can promote LC proliferation via cytokines, while LCs may influence DETC activation through antigen presentation and costimulatory signals (*10*). However, the regulatory cues governing the postnatal differentiation of DETCs and LCs, as well as the longitudinal dynamics of this process, remain poorly understood. In particular, it is still unclear whether their postnatal development is guided by shared or distinct signaling pathways and how these cell types interact in this critical period of life. Whether such cross-talk also contributes to their postnatal establishment and differentiation further remains largely unexplored. In addition, it remains unclear to what extent these differentiation programs are conserved in human epidermis, where invariant DETCs are absent and LCs differ in ontogeny and maintenance (*32*, *33*). A direct comparison of early developmental trajectories of epidermal γδ T cells and LCs between mouse and human has not been systematically performed.

Moreover, as both DETCs and LCs acquire immunophenotypic and functional properties around birth, it has been debated whether direct or distant (gut) colonization with commensal microbiota might influence this process, e.g., via microbial metabolites (*34*). This has been well studied in the oral mucosa, where microbiota have been shown to shape gingival LC development and contribute to myeloid heterogeneity (*35*, *36*). In contrast, the impact of microbiota on epidermal LC and DETC development remains largely elusive. Prior studies have only shown that DETC and LC frequencies in the epidermis remain largely unaltered in germfree (GF) mice, suggesting that their development may be less dependent on microbial colonization (*35*, *37*, *38*). Thus, whether and how commensal microbes modulate the postnatal development of epidermal DETCs and LCs remain to be explored.

In this study, we present a comprehensive temporal map of DETCs and LCs in the mouse epidermis, spanning from late embryogenesis through postnatal development. We delineate distinct waves of population expansion, morphological remodeling, and transcriptional programming that shape these resident immune compartments postnatally. Our study provides a transcriptome-resolved single-cell atlas of postnatal DETC and LC development in the mouse epidermis, uncovering distinct and progressive developmental trajectories. Furthermore, we show that LC differentiation can proceed independently of canonical DETCs in the epidermis, most likely rescued by the presence of αβDETCs. Our analyses further reveal that the postnatal specification of both DETCs and LCs occurs independently of microbial cues. Comparative analyses between mouse and human epidermal γδ T cells and LCs demonstrate conserved differentiation programs and key interaction axes across epidermal development. Together, our study reveals coordinated postnatal maturation of DETCs and LCs and demonstrates that intercellular signaling within the epidermal niche can be functionally buffered by compensatory αβDETCs. These findings further define conserved principles governing postnatal immune maturation in mouse and human epidermis.

## RESULTS

### DETCs and LCs display distinct postnatal expansion and morphological maturation programs

To understand the patterning dynamics of DETCs and LCs across development, we performed high-resolution whole-mount imaging of mouse epidermis from E17.5 to postnatal day 60 (P60) (Fig. 1A). As previously demonstrated, LCs dynamically regulate marker expression during postnatal development. While they strongly express the chemokine receptor CX3CR1 during the fetal and early postnatal period, this expression declines over time, concurrent with the progressive acquisition of differentiation markers such as epithelial cell adhesion molecule (EpCAM), Langerin (CD207), and major histocompatibility complex class II (MHC-II; Fig. 1B and fig. S1A) (*20*). In contrast, DETCs continuously express CX3CR1 and CD3ε throughout life. Because both DETCs and LCs express CX3CR1 during the fetal and first postnatal week, we used the *Cx3cr1*^GFP^ reporter line in combination with CD3ε staining to discriminate both populations. Notably, LCs expressed low levels of Langerin until P4, which was subsequently used as a definitive marker for their identification and quantification (Fig. 1B and fig. S1A). At E17.5, both DETCs and LCs were present in low numbers in the epidermis and exhibited rounded, immature morphologies (Fig. 1, B and C, and fig. S1A). By P0, LCs showed an expansion in cell number, while DETC numbers remained low (Fig. 1, B and C). Already at this early stage, LCs started forming initial protrusions (Fig. 1B). Between P1 and P7, LCs further extended dendritic processes and exhibited increased ramification (Fig. 1B and fig. S1A). In contrast, DETCs developed their characteristic dendritic morphology more gradually, with prominent ramification observed only by P30 (Fig. 1B and fig. S1A). Quantification of cell numbers over time revealed that DETCs expanded steadily, reaching a plateau around P7, and maintained stable cell numbers in the epidermis into adulthood (Fig. 1C). LCs, in contrast, displayed a more dynamic expansion pattern characterized by two waves: an initial increase around birth, a short plateau phase, followed by a pronounced proliferative burst at P7, and a subsequent decline until reaching adult equilibrium (Fig. 1C). By P60, DETCs and LCs converged to comparable densities within the epidermis (Fig. 1C).

**Fig. 1.**
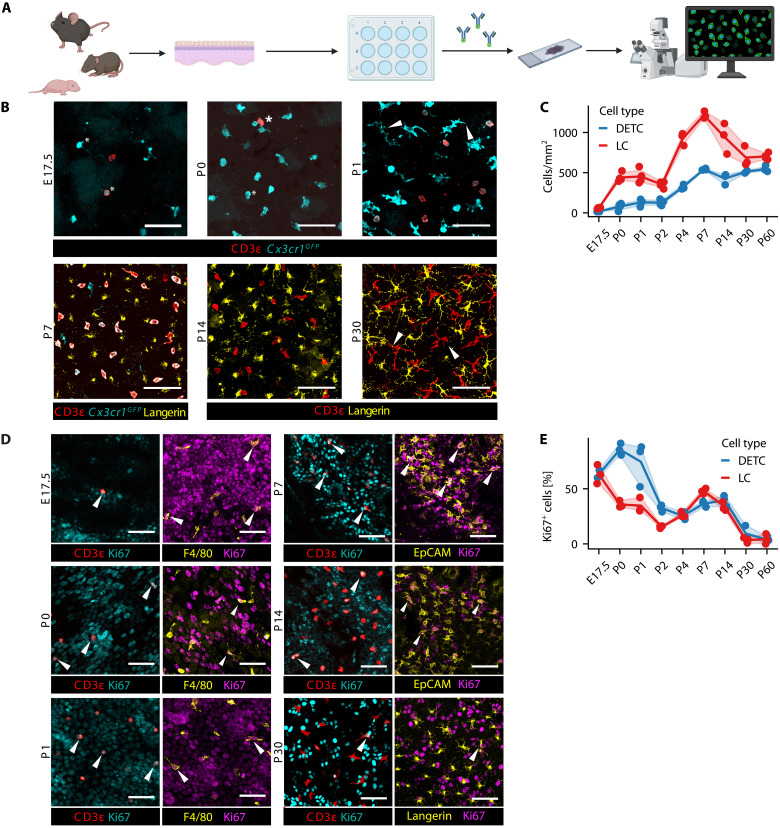
DETCs and LCs follow distinct expansion dynamics during development. (**A**) Schematic diagram of experimental flow for imaging analysis. Created in BioRender. K.K. (2026); https://BioRender.com/pncbpf0. (**B**) Representative whole-mount confocal images of epidermal DETCs and LCs at different time points. CD3ε is shown in red, *Cx3cr1^GFP^* is shown in cyan, and Langerin is shown in yellow. Asterisks indicate round cell states. Arrowheads indicate cell branching. Maximum projection of the confocal Z-stack is shown. Scale bar, 50 μm. (**C**) Quantification of DETCs (blue) and LCs (red) across developmental stages. Each dot represents one animal. Results are representative of three epidermal regions of one sample per mouse (*n* = 3 animals per group). (**D**) Representative whole-mount confocal images of epidermal Ki67^+^ DETCs and LCs at different time points. CD3e is shown in red; Ki67 is shown in cyan or magenta; and F4/80, EpCAM, or Langerin are shown in yellow. Arrowheads indicate proliferating DETCs or LCs. Selected *z* layers are shown for illustration purposes. Scale bars, 50 μm. (**E**) Percentage of Ki67^+^ DETCs (blue) and LCs (red) across developmental stages. Each dot represents one animal. Results are representative of three epidermal regions of one sample per mouse (*n* = 3 animals per group).

To assess the underlying proliferative dynamics of DETCs and LCs during this period, we labeled cycling cells using Ki67, combined with CD3ε for DETCs and F4/80 (E17.5 to P4), EpCAM (P7 to P14), or Langerin (P21 to P60) for LCs (Fig. 1D and fig. S1B). At E17.5, progenitors of both DETCs and LCs were highly proliferative, with ∼60% of cells in each compartment being Ki67^+^ (LCs: 63.17 ± 8.54% and DETCs: 63.06 ± 3.37%) (Fig. 1, D and E). By P0, LC proliferation rate declined (35.77 ± 3.13%), while nearly all DETCs remained Ki67^+^ (85.67 ± 5.29%), marking a proliferative peak for DETCs that persisted through the early postnatal days. At P7, LC proliferation increased again to 47.8%, whereas Ki67^+^ DETCs decreased to 36.64%, suggesting that DETCs were transitioning into a quiescent state, whereas LCs were entering their second proliferative wave (Fig. 1, D and E). In adulthood, only a small fraction of cells from either population remained Ki67^+^ (Fig. 1, D and E).

Together, these data show that DETCs and LCs undergo temporally distinct programs of proliferation and morphological remodeling during postnatal development. DETCs and LCs acquire ramification, proliferative capacity, and cell density in a stepwise and differential manner, indicating that each population follows a distinct expansion pattern within the developing epidermal niche.

### Temporal single-cell profiling reveals the dynamics of the epidermal immune compartment

To gain deeper insights into the molecular programs governing DETC and LC maturation, we performed single-cell RNA sequencing (scRNA-seq) of mouse epidermal immune compartment across seven developmental stages from E18.5 to P100 (Fig. 2A). Building on our imaging-based characterization, we hypothesized that a transcriptomic approach would allow us to capture dynamic changes in gene expression at single-cell resolution. We isolated single-cell suspensions from ventral epidermal sheets at early developmental stages (E18.5 to P7) and from ear epidermal sheets at later stages (P21 to P100). CD45^+^ Gr1^−^ CD19^−^ cells were sorted using fluorescence-activated cell sorting (FACS) and subjected to scRNA-seq using the 10x Genomics platform (fig. S2A). After quality control and filtering, we obtained 51,737 high-quality cells across all stages (Fig. 2B and fig. S2B). Unsupervised clustering of transcriptomes revealed 18 transcriptionally distinct clusters that distributed across the full developmental timeline (Fig. 2, B and C, and data file S1).

**Fig. 2.**
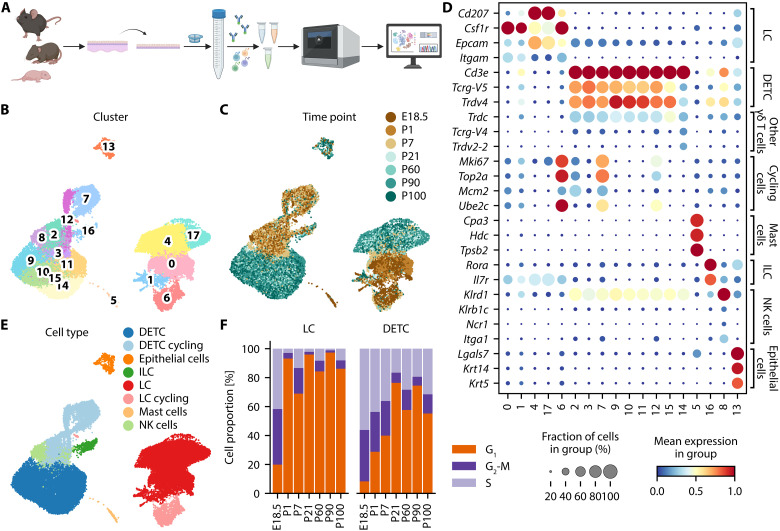
Single-cell transcriptomic profiling reveals distinct DETC and LC subsets during postnatal development. (**A**) Schematic diagram of experimental flow for scRNA-seq. Created in BioRender. K.K. (2026); https://BioRender.com/pncbpf0. (**B**) Uniform manifold approximation and projection (UMAP) plot of cell clusters after unsupervised clustering. Each color represents one cell cluster [E18.5 (*n* = 3 litters), P1 (*n* = 3 pools; three, three, and two mice per pool), and P7 to P100 (*n* = 3 per time point); *N* = 51737 cells]. (**C**) UMAP plot illustrating the distribution of single cells according to their time point of origin. (**D**) Dot plot showing key marker gene expression of cell types in the mouse epidermis across 18 identified clusters. Color represents the scaled mean expression of the gene in the respective cluster. Dot size represents the fraction of cells in the cluster expressing the gene. (**E**) UMAP plot displaying annotated immune cell types based on canonical marker gene expression. Each color represents one cell type. (**F**) Stacked bar plot illustrating the distribution of LCs (left) and DETCs (right) across G_1_ (orange), S (light purple), and G_2_-M (purple) cell cycle phases at each time point. The proportion of cells in each cell cycle phase is shown as a percentage of the total cell population.

Cell type annotation was performed using manually curated signature genes for skin-resident immune and nonimmune populations (Fig. 2, D and E). Clusters 0, 1, 4, 6, and 17 expressed *Csf1r* and *Itgam* (encoding CD11b), consistent with a macrophage/LC identity. Among these, clusters 4 and 17 showed expression of LC markers *Cd207* and *Epcam*, which were largely absent in the other LC clusters (Fig. 2D). Cluster 6 was enriched for cell cycle–associated genes such as *Mki67* and *Top2a*, indicating a highly proliferative subset within the LC compartment (Fig. 2D). Clusters 2, 3, 7, 9, 10, 11, 12, 14, and 15 expressed *Cd3e* and *Trdc*, consistent with γδ T cell identity (Fig. 2D). All clusters except cluster 14 expressed *Tcrg-V5* and *Trdv4*, identifying them as canonical Vγ5^+^Vδ1^+^ DETCs (Fig. 2D). In contrast, cluster 14 expressed *Tcrg-V4* and *Trdv2-2*, indicating that these are Vγ4^+^ γδ T cells (Fig. 2D). Additional immune populations were also identified. Cluster 5 expressed *Cpa3*, consistent with mast cells, whereas clusters 16 and 8 were annotated as innate lymphoid cells (ILCs) and natural killer (NK) cells, respectively, based on expression of *Rora* and *Il7r* (ILCs) and *Ncr1* and *Klrb1c* (NK cells). Cluster 13 showed high expression of *Epcam*, *Lgals7, Krt14*, and *Krt5*, indicating minor epithelial/keratinocyte contamination (Fig. 2D) (*39*).

To characterize proliferative states during development, we performed cell cycle phase scoring using gene signatures specific for G_1_, S, and G_2_-M phases across all annotated DETCs and LCs (Fig. 2F and data file S2). Consistent with our imaging data, we observed a sharp transition from proliferative to quiescent states in both populations between birth and adulthood. Notably, LCs exhibited two distinct proliferative peaks: first at E18.5, with 38.4% of cells in G_2_-M phase and 41.7% in S phase, and a second at P7, with 17.7% of cells in G_2_-M and 13.3% in S phase (Fig. 2F). In contrast, DETCs showed a gradual decline in proliferation, with decreasing frequencies in both S and G_2_-M phases from E18.5 to P21 (Fig. 2F). At later time points, both DETCs and LCs were predominantly in the G_1_ phase, consistent with entry into a quiescent state.

Together, these data confirm the presence of transcriptionally distinct DETC and LC cell states across development and reveal discrete waves of cell cycle activity. These findings illustrate that DETCs and LCs follow temporally and molecularly distinct differentiation trajectories in the epidermis.

### DETCs follow a linear maturation trajectory in the epidermis

Next, we focused on DETCs to characterize their transcriptional dynamics during development. Reclustering of annotated DETCs within the scRNA-seq dataset identified eight transcriptionally distinct DETC clusters (Fig. 3A and data file S3). Clusters 0, 1, and 2 were enriched at early stages (E18.5 to P7), whereas clusters 3 to 7 were predominantly composed of cells from later time points (P21 to P100), with cluster 3 being particularly overrepresented at P21 (Fig. 3, B and C). All clusters robustly expressed canonical DETC–associated genes, including *Il2rb* (CD122) and *Il2rg* (CD132), encoding IL-15 receptor subunits critical for DETC proliferation and survival, as well as *Thy1*, *Xcl1*, and *Klrk1* (encoding NKG2D) (Fig. 3D) (*40*–*43*). Key transcription factors involved in DETC development, including *Runx3* and *Ets1*, were also expressed across all clusters, albeit at lower levels (Fig. 3D) (*44*, *45*). Clusters 0, 1, and 2 showed high expression of cell cycle–associated genes such as *Mki67*, *Top2a*, *Mcm2*, and *Mcm6*, indicating highly proliferative states during fetal and early postnatal development, consistent with our imaging data (Fig. 3D). In contrast, clusters 3 to 7, which were enriched in adult epidermis, expressed higher levels of T cell activation–related genes including *Nr4a2* and *Fos* (Fig. 3D). Notably, DETCs up-regulated the growth factor *Areg* in clusters 4 to 7, which were abundant after P21, suggesting the acquisition of tissue-repair functions during adulthood (Fig. 3D) (*46*). Moreover, clusters 4 to 7 also expressed genes with currently unknown roles in γδ T cell biology, such as *Ramp3*, *Gem*, and *Mest* (mesoderm-specific transcript), with cluster 5 showing the highest expression of all three. Next, we characterized distinct transcriptional programs in DETCs at different developmental stages using published gene sets (Fig. 3E and data file S4). This analysis revealed progressive up-regulation of gene signatures related to structural and morphological remodeling, such as dendrite development (GO:0016358) and cell projection organization (GO:0030030), as well as tissue repair programs (*47*, *48*). Dendrite development and cell projection organization signatures increased gradually over time, whereas tissue repair programs were prominently induced from P60 onward, coinciding with adulthood (Fig. 3E).

**Fig. 3.**
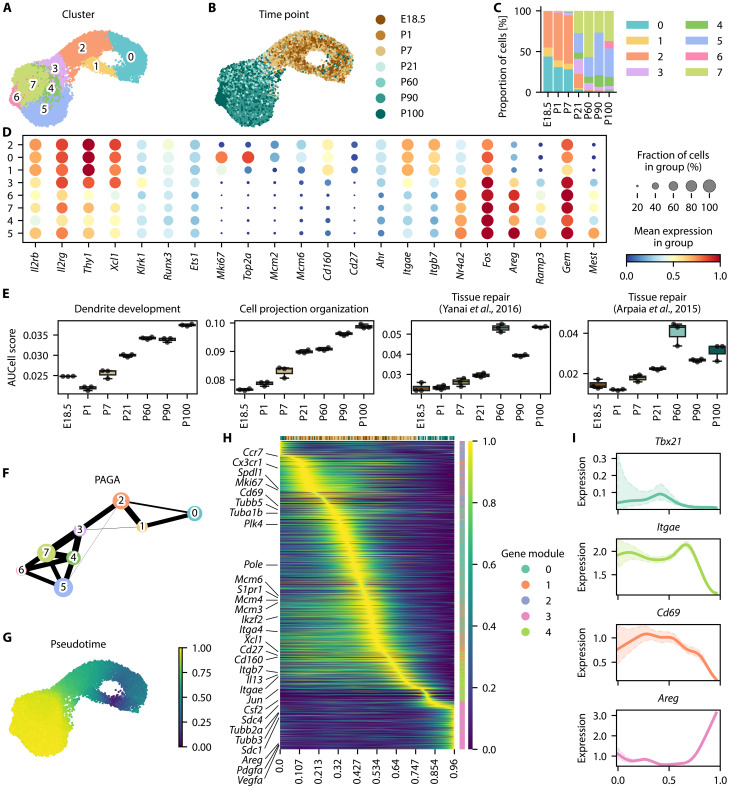
DETCs gradually acquire of tissue specificity and maturation markers in the developing epidermis. (**A**) UMAP plot of reclustered DETCs. Eight clusters were identified (*N* = 25,911 cells). Colors represent individual clusters. (**B**) UMAP plot illustrating the distribution of cells according to their time point of origin. Each color represents one analysis time point. (**C**) Bar plot depicting the proportion of cells assigned to each cluster across the different time points. (**D**) Dot plot showing expression of selected genes across DETC clusters. Color represents the scaled mean gene expression, and dot size represents the fraction of cells expressing the gene. (**E**) Box plot showing mean AUCell score for Gene Ontology (GO) terms and gene sets from previously published data in DETCs across different time points (*47*, *48*). Dots represent individual biological samples. (**F**) Partition-based graph abstraction (PAGA) graph at cluster resolution. Each node represents one cluster, with node size proportional to the number of cells in the cluster. Edges indicate connectivity between clusters. Edge thickness reflects transcriptomic similarities between clusters. (**G**) UMAP plot colored by diffusion pseudotime values calculated on the basis of PAGA graph with the starting cell picked on the basis of predicted initial state (fig. S3A). Color scale is shown in legend. (**H**) Heatmap showing the smoothed expression of top 2000 highly variable genes ordered by their peak of expression along pseudotime (left to right) and organized into modules (top to bottom). Rows represent genes. Columns depict pseudotime progression. Gene modules were identified on the basis of expression patterns along pseudotime. Scale reflects the smoothed and scaled expression value of the genes. (**I**) Graphs showing the expression profile of individual, key genes along pseudotime from early to late stages (left to right). Colors indicate the respective module membership. Smoothed gene expressions are shown.

Next, we applied CellRank to model DETC lineage progression based on our temporally annotated scRNA-seq dataset (*49*). We first constructed a real-time kernel using experimental time points to infer initial and terminal states of DETC differentiation (fig. S3, A and B). We then computed diffusion pseudotime trajectories (DPT) along a partition-based graph abstraction (PAGA) at the cluster level, enabling modeling of gene expression changes over pseudotime (Fig. 3, F and G) (*50*). To identify coordinated transcriptional programs, we smoothed gene expression along DPT and clustered the resulting expression trajectories into five distinct gene modules (Fig. 3, H and I, fig. S3C, and data file S5). Gene modules 0 and 2, enriched at early to intermediate pseudotime, contained genes associated with S phase and G_2_-M phase of the cell cycle and DNA replication, including *Mcm3*, *Mcm4*, *Mcm6*, *Pole*, *Tuba1b*, *Tubb5*, *Mki67*, *Sdl1*, and *Plk4* (Fig. 3H). Gene module 1, expressed predominantly at early stages (until P7), was enriched in genes associated with IFN-γ–producing γδ T cell lineages, such as *Cd27* and *Cd160*, possibly reflecting residual thymic programming (Fig. 3H). This finding aligns with the enhanced IFN-γ production reported in neonatal DETCs compared to IL-13–biased adult DETCs (*51*). Module 1 also contained genes linked to T cell activation and migration, including *Cd69*, *Ccr7*, *Cx3cr1*, *Itga4*, and *S1pr1*, supporting the idea that DETCs retain migratory and activation signatures perinatally before transitioning to tissue residency (Fig. 3, H and I) (*52*). Gene module 4 showed a similar temporal pattern to module 1-high at early stages but declining more gradually (fig. S3C). This module included genes that remained consistently expressed until adulthood, such as *Itgae*, *Itgb7*, *Csf2*, *Il13*, *Xcl1*, and *Jun*, suggesting roles in tissue anchoring, cytokine production, and baseline effector functions (Fig. 3, H and I). Last, gene module 3, uniquely up-regulated in differentiated DETCs, was enriched for genes associated with tissue repair (*Areg*, *Vegfa*, and *Pdgfa*) and cytoskeletal remodeling (*Tubb3*, *Tubb2a*, *Sdc1*, and *Sdc4*), potentially contributing to the acquisition of dendritic morphology and repair capacity of mature DETCs (Fig. 3, H and I). Together, our analysis identifies a linear differentiation trajectory of DETCs in the epidermis, characterized by a gradual loss of migratory and thymic features and the progressive acquisition of tissue repair–associated programs.

### LCs undergo stepwise and programmed differentiation in the epidermis

To dissect the differentiation dynamics of LCs during perinatal development, we reclustered all previously annotated LC single-cell transcriptomes (e.g., *Cd207*, *Csf1r*, *Epcam*, and *Itgam*). Unbiased clustering revealed 11 transcriptionally distinct LC clusters across the developmental time course (Fig. 4, A and B). Clusters 0 to 5 were enriched in cells from fetal and early postnatal stages (E18.5 to P7), while clusters 6 to 10 were dominated by cells from later stages (P21 to P100) (Fig. 4, B and C). At E18.5 and P1, clusters 0 to 2 were the most prevalent, but by P7, their frequencies markedly declined, coinciding with the emergence of later-stage clusters. Thus, P7 marks a key developmental transition in LC transcriptional states. To characterize these clusters, we identified differentially expressed genes (DEGs) and gene sets, revealing distinct transcriptional programs (fig. S4, A and B, and data file S6). Early-stage clusters (0 to 2 and 5) expressed high levels of macrophage progenitor genes, including *Cx3cr1*, *Adgre1*, *Fcgr1*, and *Mrc1* (Fig. 4D). Notably, these clusters also showed expression of the microglial identity genes *Hexb*, *Apoe*, *Trem2*, *P2ry12*, and *Tmem119.* Thus early LC progenitors in the epidermis are transcriptionally reminiscent of fetal microglia-like progenitors recently reported in human epidermis, testis, and heart (Fig. 4D) (*53*). These clusters also expressed key transcription factors such as *Id2*, *Spi1*, *Irf8*, and *Cebpb*. While *Id2*, *Spi1*, and *Irf8* promote LC differentiation, *Cebpb* has been implicated as a negative regulator (*54*–*57*). Complement system genes were prominently expressed in early clusters and rapidly declined by P7, suggesting roles in perinatal LC function. Core LC-maintenance genes such as *Csf1r* and *Tgfbr1* were expressed in all clusters but were elevated in early stages (clusters 0 to 5), accompanied by *Tgfb1* expression (Fig. 4D). In contrast, *Il34* was undetectable, and *Csf1* was transiently expressed only in early LC progenitors, indicating that these niche factors are likely provided by other skin-resident cells (fig. S4, C and D). The expression of *Csf2ra* and *Csf2rb* across all clusters suggests that LCs remain poised to respond to CSF2 [also called granulocyte-macrophage CSF (GM-CSF)] (Fig. 4D). Clusters 3, 4, and 6 to 10, enriched in later developmental stages, expressed hallmark LC markers, including MHC-II genes (e.g., *H2-Aa* and *H2-Eb1*), *Cd207*, and *Epcam* (Fig. 4D).

**Fig. 4.**
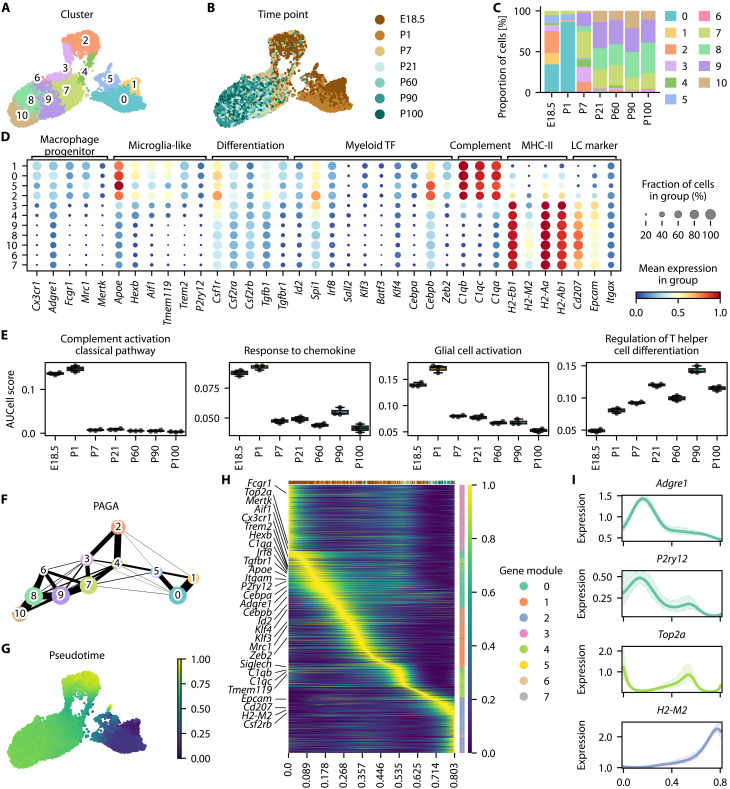
Stepwise maturation of LC in the developing epidermis. (**A**) UMAP plot of reclustered LC. Eleven clusters were identified (*N* = 17,740 cells). Colors represent individual clusters. (**B**) UMAP plot illustrating the distribution of cells according to their time point of origin. Each color represents one analysis time point. (**C**) Bar plot depicting the proportion of cells assigned to each cluster across the different time points. (**D**) Dot plot showing expression of selected genes across the different clusters. Color represents the scaled mean gene expression, and dot size represents the fraction of cells expressing the gene. TF, transcription factors. (**E**) Box plot showing mean AUCell scores per replicate of selected GO terms across the different time points. Dots represent individual biological samples. (**F**) PAGA graph at cluster resolution. Each color represents one cell cluster [see also (A)]. Each node represents one cluster, with node size proportional to the number of cells in the cluster. Edges indicate connectivity between clusters. Edge thickness reflects transcriptomic similarities between clusters. (**G**) UMAP plot colored by diffusion pseudotime values calculated on the basis of PAGA graph using predicted initial state as starting cell (fig. S3E). Color scale is shown in legend. (**H**) Heatmap showing the smoothed expression of top 2000 highly variable genes ordered by their peak of expression along pseudotime (left to right) and organized into modules (top to bottom). Rows represent genes. Columns depict pseudotime progression. Gene modules were identified on the basis of expression patterns along pseudotime. Scale reflects the smoothed and scaled expression value of the genes. (**I**) Graphs showing the expression profile of individual, key genes along pseudotime from early to late stages (left to right). Colors indicate the respective module membership. Smoothed gene expressions are shown.

To better understand functional transitions across development, we performed Gene Ontology (GO) term enrichment analyses. At E18.5 and P1, early LCs were enriched for terms such as complement activation classical pathway (GO:0006958), response to chemokine (GO:1990868), and leukocyte activation involved in inflammatory response (GO:0002269) (Fig. 4E and fig. S4B). These signatures, alongside enrichment for glial activation (GO:0061900), reinforce the activated and microglia-like signature of LC progenitors. GO terms related to DNA replication (DNA replication initiation; GO:0006270) were also enriched at E18.5 and P7, consistent with our imaging data showing proliferative bursts at these time points (fig. S4B). Genes linked to regulation of T helper cell differentiation were up-regulated from P1 onward, suggesting a potential role for LCs in influencing DETC polarization in the early epidermis (Fig. 4E). In contrast, later stages were enriched for terms such as cell-cell junction assembly (GO:0007043), indicating increasing involvement in structural organization and barrier function (fig. S4B). Together, these data reveal a stepwise LC differentiation process during postnatal development, transitioning from a progenitor and microglia-like transcriptional program before P7 to a mature, tissue-integrated LC identity after P21. This is accompanied by two proliferative bursts—after epidermal colonization (E18.5) and at P7. Pseudotime analysis using PAGA and DPT, with cluster 1 at E18.5 as the root, revealed a bifurcated trajectory: one branch toward cluster 10 (dominant at later stages) and another toward proliferative cluster 2 (Fig. 4, F and G, and fig. S4E). Cluster 2 thus represents a key branching point that diverts cells from differentiation into a proliferative loop, which ultimately feeds into fully differentiated clusters (*7*–*10*). Our analysis does not exclude the possibility that mature LCs reenter the cycle via this proliferative route, supporting the idea of intermittent expansion during LC differentiation. We further defined gene modules across pseudotime (fig. S4F and data file S7). Modules 0 and 3, active early, contained macrophage progenitor and microglial identity genes (e.g., *Fcgr1*, *Adgre1*, *P2ry12*, *Hexb*, and *Tmem119*) (Fig. 4, H and I). Module 4, which peaked at early and intermediate stages, comprised proliferation-related genes such as *Top2a*. Module 2, enriched at the terminal trajectory, contained canonical LC markers (*Cd207*, *H2-M2*, and *Epcam*), confirming the acquisition of a mature LC identity over time.

Collectively, our results support a model in which LC development progresses through discrete transcriptional states defined by successive functional modules, beginning with a macrophage progenitor identity and a microglia-like signature and culminating in fully differentiated LCs. This trajectory is punctuated by temporally regulated proliferative bursts.

### LC development proceeds independently of canonical DETCs

To assess whether DETCs influence LC development in the perinatal epidermis, we used a computational cell-cell communication inference strategy using LIANA and Tensor-cell2cell (*58*). Analyses were performed independently for each biological replicate across developmental time points, integrating predictions from multiple ligand-receptor inference methods into a unified consensus score. These were compiled into a four-dimensional (4D) tensor (sender cell, receiver cell, ligand-receptor pair, and developmental context), followed by tensor decomposition, which revealed seven communication factors (fig. S5A and data file S8). Among these, factors 1, 5, 6, and 7 reflected prominent DETC-LC interactions across development (Fig. 5A). While factors 5 and 7 represented predicted signals enriched in early development, factors 1 and 6 were predominant at later stages (Fig. 5A). Notably, factors 1 and 6 included predicted LC-to-DETC signals at both early and late time points (Fig. 5, A and B). In addition to adhesion-related interactions, a strong prediction for *Ccl4* → *Ccr8* signaling between LCs and DETCs was found during early development (Fig. 5B). However, CCL4 is not known to functionally bind CCR8, questioning the relevance of this interaction. Conversely, factors 6 and 7 captured DETC-derived signals to LCs, including cytokine-receptor pairs such as *Il13* → *Il13ra*_*Il4ra*, *Csf2* → *Csf2ra*_*Csf2rb*, and *Tgfb1* → *Tgfbr1*_*Tgfbr2*. These pathways are connected to LC biology but have not been previously attributed to DETCs during development (Fig. 5B) (*59*–*62*). The *Tgfb1* → *Tgfbr1*_*Tgfbr2* interaction scored highly during early stages and waned over time. These results suggest that DETCs may provide developmental cues for LC development in the early epidermis.

**Fig. 5.**
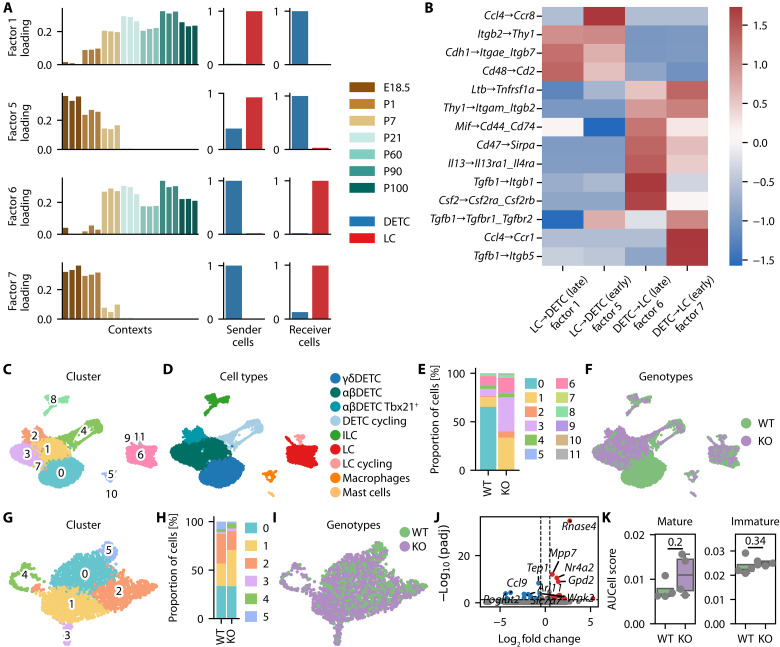
Loss of canonical DETCs during postnatal development does not affect the transcriptomic profile of adult LCs. (**A**) Graphs depicting results of running LIANA and Tensor-cell2cell frameworks. Factor loadings (*y* axis) are displayed for each element of a given dimension. (**B**) Heatmap showing the loadings of selected interactions in factors 1 and 5 to 7. Scale represents the loadings for an interaction in the factor. (**C**) UMAP plot of cell clusters after unsupervised clustering in *Tcrd^−/−^* [knockout (KO)] (*n* = 4) and *Tcrd^+/+^* [wild-type (WT)] (*n* = 4) animals. Twelve clusters were identified (*N* = 20,632 cells). (**D**) UMAP plot showing different annotated cell types and cycling cells based on core marker expression. (**E**) Bar plot showing the proportion of cells assigned to each cluster in KO and WT animals. (**F**) UMAP plot showing cells colored by genotype of origin. Cells from KO mice are shown in purple, and cells from WT mice are shown in green. (**G**) UMAP plot of reclustered LC. Six clusters were identified (*N* = 2606 cells). (**H**) Bar plot showing the proportion of cells assigned to each cluster originating from either KO or WT animals. (**I**) UMAP plot showing cells colored by genotype. (**J**) Volcano plot of DEGs in LCs from KO versus WT mice as determined using PyDESeq2 (pseudobulked gene expression). Vertical lines mark a log_2_ fold change of 0.5 (fold change of ∼±1.41), and horizontal line represents a –log_10_ *P* value of 1.3 (*P* value of 0.05). *P* adj, adjusted *P*. (**K**) Box plots of mean AUCell scores of gene sets for mature and immature LC (signatures derived from Fig. 4) in WT and KO mice (*n* = 4). Dots represent individual biological replicates. Statistical testing was performed using Mann-Whitney *U* rank test.

To test whether DETCs are functionally required for LC development, we analyzed *Tcrd*-deficient (*Tcrd*^−/−^) mice, which lack all γδ T cells (*63*). In these mice, DETCs are replaced by compensatory αβ T cells (hereafter αβDETCs) (*64*). We performed scRNA-seq on epidermal immune cells from adult *Tcrd*^+/+^ and *Tcrd*^−/−^ mice (fig. S5B). As expected, *Tcrd*^−/−^ mice lacked γδDETCs (cluster 0) but harbored abundant αβDETCs (cluster 1), whereas ILCs, mast cells, and macrophages were equally present in both genotypes (Fig. 5, C to F, and data file S9). Next, we subclustered LCs and identified six transcriptionally distinct clusters that were shared across both genotypes without genotype-specific alterations (Fig. 5, G to I). Pseudobulk differential expression analysis using PyDESeq2 (accounting for batch effects) revealed only 66 significant DEGs (adjusted *P* < 0.05) between *Tcrd*^+^^/^^+^ and *Tcrd*^−^^/^^−^ LCs, with modest fold changes (Fig. 5J and data file S10). While some of these genes were associated with LC or dendritic cell maturation, they did not indicate a substantial disruption of developmental programs. To quantify LC maturation, we constructed gene signatures for immature and mature LCs based on our time-course dataset and computed maturity scores (data file S11). These scores did not differ significantly between genotypes (Fig. 5K), suggesting that γδDETCs are not essential for LC maturation.

To further explore whether αβDETCs functionally compensated for absent γδDETCs, we reapplied LIANA and Tensor-cell2cell using genotype as the contextual variable and modeled interactions among γδDETCs, αβDETCs, and LCs. Four communication factors were identified, yet none displayed genotype-specific signaling patterns (Fig. 6, A and B, and data file S12). Predicted interactions such as *Il13* → *Il13ra_Il4ra*, *Csf2* → *Csf2ra*_*Csf2rb*, and *Tgfb1* → *Tgfbr1*_*Tgfbr2* were shared between γδDETCs and αβDETCs, with comparable interaction scores across genotypes (Fig. 6C). Consistent with these interaction predictions, transcript-level analysis revealed largely overlapping cytokine expression profiles between adult γδDETCs and αβDETCs (Fig. 6D and data file S13). While *Csf2* expression was reduced and *Il13* expression increased in the absence of γδDETCs, *Tgfb1* levels remained unchanged (Fig. 6, D and E). Of note, the analysis included adult cells only and might not fully reflect compensation during earlier time points. LCs maintained stable expression of the corresponding receptors (*Csf2ra/Csf2rb*, *Il13ra1/Il4ra*, and *Tgfbr1/Tgfbr2*) (Fig. 6F), and downstream pathway activity scores for CSF2, IL-13, and TGF-β signaling in LCs were not significantly altered between genotypes (Fig. 6G), pointing to a proficient induction of these pathways during LC maturation.

**Fig. 6.**
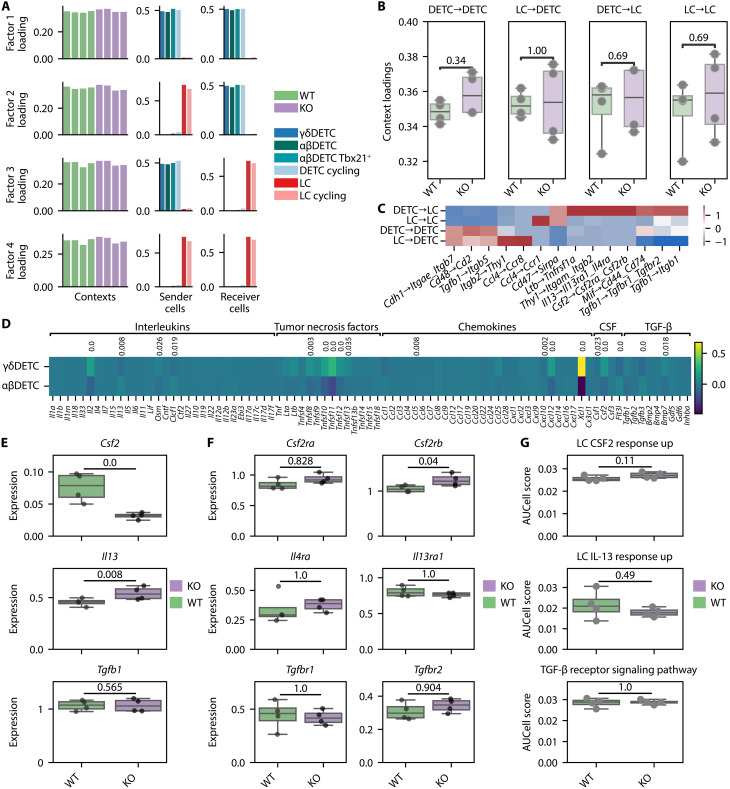
αβDETCs functionally compensate the loss of canonical DETCs in adult *Tcrd*^−/−^ mice. (**A**) Graphs depicting results of running LIANA and Tensor-cell2cell frameworks. Each row represents a factor displaying paracrine signaling between DETCs and LCs (colors depicted in the legend) and each column a tensor dimension, wherein each bar plot represents an element of that dimension (genotype, a sender cell, or a receiver cell). Factor loadings (*y* axis) are displayed for each element of a given dimension. Colors of individual genotypes are depicted in legend. (**B**) Box plots of context loading separated by genotype [KO (purple) versus WT (green)] for possible cell-cell interactions derived from tensor decomposition. Dots represent individual biological samples. Statistical significance was assessed using the Mann-Whitney *U* rank test, with *P* values corrected for multiple testing using the Benjamini-Hochberg procedure. (**C**) Heatmap showing the loadings of selected interactions in possible cell-cell interactions. Scale represents the loadings for an interaction in the communication pathway. (**D**) Heatmap showing the expression of selected signaling molecules in αβDETCs versus γδDETCs. Scale represents the expression values. (**E**) Box plots of mean expression of *Csf2*, *Il13*, and *Tgfb1* in KO versus WT (*n* = 4). Dots represent individual biological replicates. Statistical testing was performed using Mann-Whitney *U* rank test. (**F**) Box plots of mean expression of *Csf2ra*, *Csf2rb*, *Il4ra*, *Il13ra1*, *Tgfbr1*, and *Tgfbr2* in KO versus WT (*n* = 4). Dots represent individual biological replicates. Statistical testing was performed using Mann-Whitney *U* rank test. (**G**) Box plots of mean AUCell scores of LC signaling responses downstream of CSF2, IL-13, and TGF-β pathways for KO versus WT (*n* = 4). Dots represent individual biological replicates. Statistical testing was performed using Mann-Whitney *U* rank test.

Together, these findings indicate that DETCs may contribute to a cytokine-rich microenvironment conducive to LC development. However, αβDETCs can recapitulate key signaling features of bona fide γδDETCs and may support LC differentiation in their absence.

### Commensal microbiota are dispensable for DETC and LC maturation

As the skin is among the first organs to be colonized by commensal microorganisms, we next asked whether the microbiota influence the postnatal maturation of DETCs and LCs. To address this, we studied GF mice, which are devoid of microbial colonization, and wildlings, natural microbiota-based mouse model, which harbors a complex and naturally coevolved microbiota and fungi resembling that of wild mouse counterparts (*65*, *66*). We profiled epidermal immune cells at early postnatal (P1 and P7) and adult (P90 and P100) stages in specific pathogen–free (SPF), GF, and wildlings to assess both immediate and long-term microbiota-driven effects (Fig. 7, A and B, and fig. S6, A and B). DETCs and LCs were annotated on the basis of previously defined gene signatures from our time-course dataset. Immature DETCs were identified by *Cd160*, *Itgae*, and *Itgb7*, and mature DETCs by *Areg*, *Ramp3*, and *Mest*. Immature LCs were marked by complement-associated genes (*C1qb*, *C1qc*) and microglia-related transcripts (*Trem2*), while mature LCs expressed *Cd207*, *Epcam*, and MHC-II genes including *H2-Eb1*. Cycling DETCs and LCs were defined by proliferation-associated genes such as *Mki67* and *Top2a* (Fig. 7C, fig. S6, C and D, and data file S14).

**Fig. 7.**
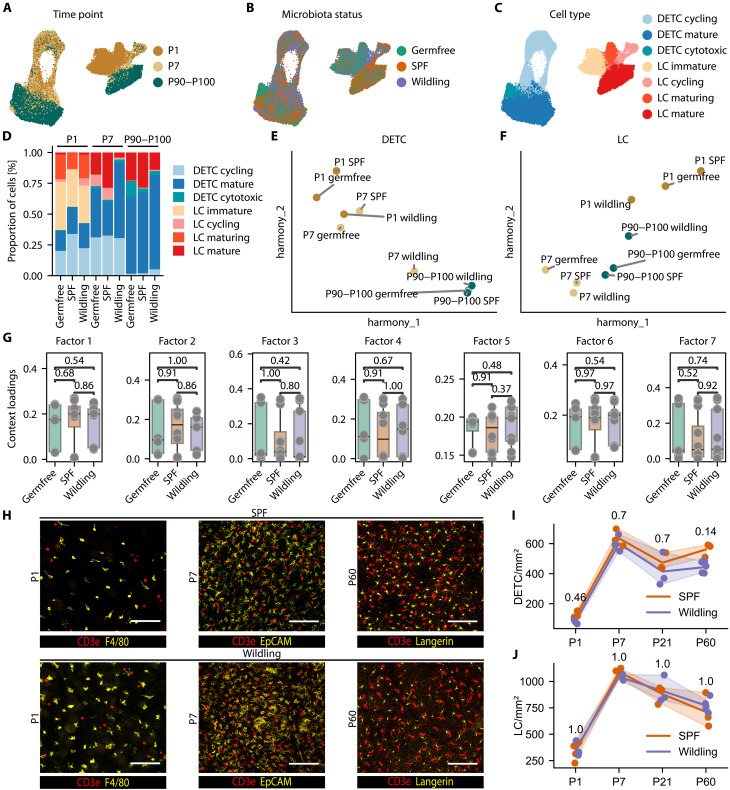
Absence or higher complexity of commensal microbiota does not affect DETC and LC maturation. (**A**) UMAP plot of cells from GF mice (*n* = 3), SPF mice (*n* = 3), and wildlings (*n* = 3) (*N* = 80,556 cells) colored by time point of origin. (**B**) UMAP plot of cells according to their microbiota status [GF (green), SPF (orange), and wildlings (purple)]. (**C**) UMAP plot colored by cell type. Annotation is based on core marker genes shown in fig. S6D. (**D**) Bar plot showing the proportion of cells within the different cell types across different microbiota statuses (GF, SPF, and wildlings) and analyzed time points. Time points are indicated above bar plot. Colors represent cell types. (**E** and **F**) Scatter plot showing the median of the harmony embedding for each time point and microbial status for DETCs (E) and LCs (F). Colors indicate time points. Microbial status is labeled within the scatter plot. (**G**) Box plots of the context loadings for all seven factors recovered by factor decomposition of a tensor built using microbial status and time points as contexts. (**H**) Representative images of epidermal DETCs and LCs in SPF and wildlings at P1, P7, and P60. Langerin (orange) and CD3e (red) are shown. Maximum projection of the confocal z-stack is shown. Scale bar, 50 μm. (**I** and **J**) Quantification of DETC (I) and LC (J) densities in SPF mice (orange) and wildlings (purple) across developmental stages. Each dot represents one animal. Results are representative of three epidermal regions of one sample per mouse (*n* = 3 to 5 animals per group). Statistical significance in (G), (I), and (J) was assessed using the Mann-Whitney *U* rank test, with *P* values corrected for multiple testing using the Benjamini-Hochberg procedure.

At P1, both DETCs and LCs displayed similar transcriptomic profiles across SPF, GF, and wildlings (Fig. 7D). At P7, wildlings showed a modest reduction in cell clusters associated with differentiated LCs and a corresponding increase in mature DETCs; however, this effect was no longer evident by P90 to P100 (Fig. 7D). Calculating cluster medians of the Harmony embedding confirmed that developmental time point and not microbial status was the primary driver of transcriptional variation in both cell types (Fig. 7, E and F). To further explore potential microbiota-driven alterations in cell-cell communication, we again applied the LIANA and Tensor-cell2cell frameworks. Tensor decomposition revealed seven communication factors (fig. S7A and data file S15), none of which differed significantly across microbial conditions (GF, SPF, and wildlings) (Fig. 7G). Instead, observed variation in communication patterns was entirely attributable to developmental stage (fig. S7, A and B). To validate these transcriptomic findings, we performed immunofluorescent staining of DETCs and LCs across developmental time points in SPF mice and wildlings. In line with previous reports showing comparable mucosal DETC and LC frequencies in GF and SPF animals, we found no significant differences between SPF mice and wildlings (Fig. 7, H to J) (*35*, *37*, *38*).

Together, these results indicate that although microbial colonization may exert transient effects on LC maturation during early postnatal life, both DETCs and LCs ultimately mature independently of the microbiota in the epidermis. Thus, commensal microbes are dispensable for their postnatal development under steady-state conditions.

### Core maturation programs and γδ T cell–LC signaling networks are conserved in human epidermis

Invariant Vγ5^+^Vδ1^+^ DETCs represent a mouse-specific epidermal T cell population and do not exist in humans (*67*). In addition, human LCs differ from murine LCs in ontogeny and maintenance (*8*). We therefore tested whether the maturation programs and intercellular signaling pathways identified in mouse epidermis are conserved in human epidermis during development, despite these species-specific differences. To address this question, we reanalyzed two human prenatal and adult skin scRNA-seq datasets (Fig. 8A) (*68*, *69*). Because these datasets contained multiple dermal and epidermal immune populations, we first annotated epidermal γδ T cells and LCs based on canonical marker expression before performing downstream analyses. Unsupervised clustering identified distinct clusters of fetal and adult γδ T cell populations, as well as fetal LC progenitors, fetal immature LCs, and adult mature LCs (Fig. 8, B to F, and data file S16). We observed specific stage-dependent transcriptional programs in both lineages. Similar to murine DETCs, human fetal epidermal γδ T cells preferentially expressed proliferation-associated genes and tissue-residency features, whereas their adult counterparts expressed *AREG* (Fig. 8E). Accordingly, dendrite development and tissue-repair signatures were up-regulated in adult γδ T cells compared to fetal cells (Fig. 8G and data file S17). Human fetal LC progenitors and immature LCs expressed macrophage progenitor–associated genes, complement components, and microglia-like signature genes including *APOE*, *TREM2*, and *P2RY12*, whereas adult mature LCs up-regulated MHC-II genes and regulators of T helper cell differentiation (Fig. 8, F and H, and data file S17). Complement pathway activity predominated during fetal stages, while antigen presentation and adaptive immune regulatory programs increased in adulthood. These transitions mirrored the temporal maturation patterns defined in mouse epidermis.

**Fig. 8.**
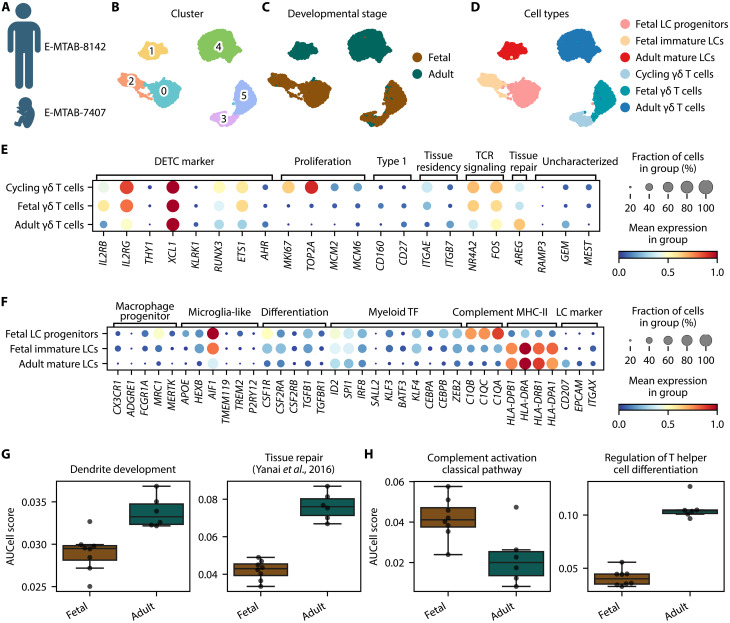
Human epidermal LC and γδ T cells show signatures similar to murine epidermal immune cells. (**A**) Schematic representation of human adult and fetal data origin (*68*, *69*). Created in BioRender. K.K. (2026); https://BioRender.com/pncbpf0. (**B**) UMAP plot of cell clusters after unsupervised clustering. Each color represents one cell cluster (*N* = 5241 cells). (**C**) UMAP plot illustrating the distribution of single cells according to their time context of origin (fetal versus adult). (**D**) UMAP plot displaying annotated immune cell types. Each color represents one cell type. (**E**) Dot plot showing key marker gene expression of γδ T cells in the human epidermis across different contexts (cycling versus fetal versus adult). Color represents the scaled mean expression of the gene in the respective context. Dot size represents the fraction of cells in the context expressing the gene. (**F**) Dot plot showing key marker gene expression of LC in the human epidermis across different contexts (fetal progenitors versus fetal immature versus adult). Color represents the scaled mean expression of the gene in the respective context. Dot size represents the fraction of cells in the context expressing the gene. (**G**) Box plot showing mean AUCell scores of selected GO terms and gene sets from previously published data (*47*, *48*) across the different contexts (fetal versus adult) in γδ T cells. Dots represent individual biological samples. (**H**) Box plot showing mean AUCell scores of selected GO terms across the different contexts (fetal versus adult) in LC. Dots represent individual biological samples.

We next tested whether γδ T cell-LC communication networks were similarly conserved. We applied LIANA and Tensor-cell2cell to model ligand-receptor interactions across fetal (“early”) and adult (“late”) contexts. This analysis identified four communication factors corresponding to early and late bidirectional signaling states between γδ T cells and LCs (Fig. 9A and data file S18). Across developmental stages, both species shared prominent interaction modules. In particular, we detected conserved cytokine signaling axes, including *IL13 → IL13RA1_IL4R*, *CSF2 → CSF2RA_CSF2RB*, and *TGFB1 → TGFBR1_TGFBR2* (Fig. 9B). Gene expression analyses confirmed stage-dependent regulation of *IL13*, *CSF2*, and *TGFB1* in human γδ T cells, while LCs maintained expression of the corresponding receptors (Fig. 9C). We also identified conserved integrin-mediated adhesion interactions (*ICAM1 → ITGAM_ITGB2*), TNF-family signaling, Notch-related interactions, and immune-regulatory axes such as *CD47 → SIRPA* and *MIF→CD74* (Fig. 9, D and E). Together, these analyses demonstrate that, despite species-specific differences in cellular composition and ontogeny, epidermal γδ T cells and LCs engage conserved mouse-to-human maturation programs and intercellular signaling networks within the developing epidermal tissue niche.

**Fig. 9.**
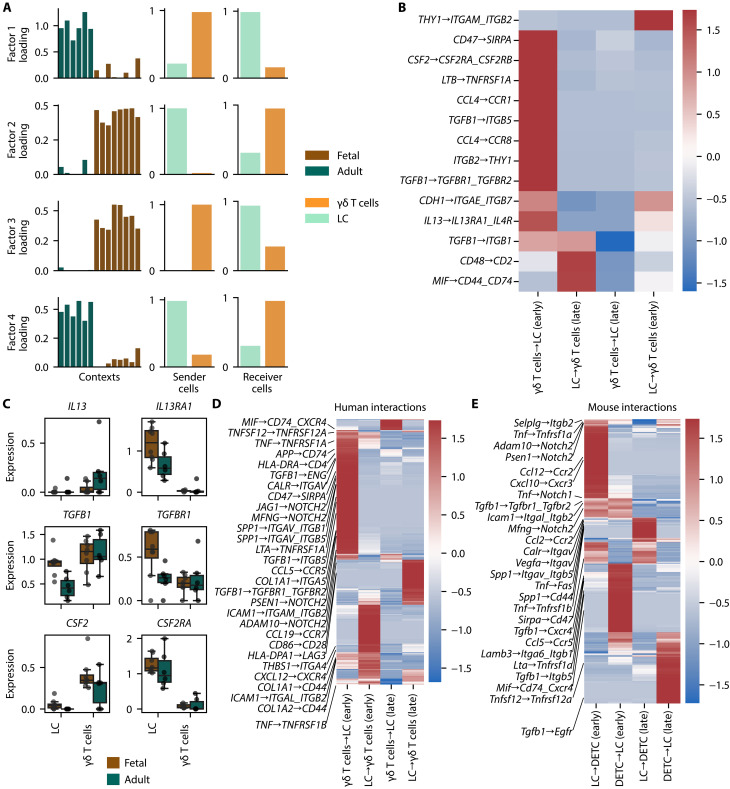
Human epidermal LCs and γδ T cells show interactions similar to murine epidermal immune cells. (**A**) Graphs depicting results of running LIANA and Tensor-cell2cell frameworks. Factor loadings (*y* axis) are displayed for each element of a given context. (**B**) Heatmap showing the loadings of selected interactions in factors 1 to 4. Scale represents the loadings for an interaction in the factor. (**C**) Box plot showing the expression of *IL13*, *TGFB1*, and *CSF2* in γδ T cells and *IL13RA1*, *TGFBR1*, and *CSF2RA* in LCs in different contexts (fetal versus adult). Dots represent individual biological replicates. Statistical testing was performed using Mann-Whitney *U* rank test. (**D**) Heatmap showing the loadings of top 500 human-specific interactions in cell-cell communication pathways as represented by factors 1 to 4 in (B). Scale represents the loadings for an interaction in the factor. Shown interactions correspond to a few shortlisted signaling pathways also detected in the mouse analysis. (**E**) Heatmap showing the loadings of top 500 mouse-specific interactions in cell-cell communication pathways as represented by factors 1 to 4 in (B). Scale represents the loadings for an interaction in the factor. Shown interactions correspond to a few shortlisted signaling pathways also detected in the human analysis.

## DISCUSSION

Our study provides a comprehensive temporal landscape of the fetal and postnatal development of DETCs and LCs in the mouse epidermis. By integrating high-resolution whole-mount imaging and single-cell transcriptomics, we delineate distinct developmental trajectories of these resident immune populations, characterized by successive waves of proliferation, morphological remodeling, and transcriptional programming. Our findings demonstrate that LC maturation can proceed independently of canonical DETCs in the presence of compensatory αβDETCs and that both cell types develop independent of commensal microbial colonization, suggesting a robust perinatal immune development program, which is partially conserved in developing human epidermis.

Our results align with previous studies demonstrating that DETC and LC progenitors colonize the mouse epidermis during the perinatal period (*19*, *23*). We detected both progenitor populations in the developing epidermis around E17.5. Thus, colonization by one cell type is not required for robust epidermal entry of the other, consistent with prior reports showing LC colonization within the same developmental window in *Rag*^*−/−*^ *γc*^*−/−*^ mice lacking DETCs (*20*). However, we observed distinct postnatal expansion dynamics for DETCs and LCs. DETCs gradually increased in density until reaching their adult network configuration, whereas LCs underwent two proliferative waves at E18.5 and P7. LC numbers peaked at P7 and subsequently declined until adulthood, a pattern also observed during postnatal development in other tissue-resident macrophage populations such as microglia in the central nervous system (CNS) parenchyma (*70*, *71*). The mechanisms underlying this initial expansion and subsequent contraction of the population size remain to be defined. We also observed differences in the timing of dendrite formation: LCs began branching earlier than DETCs, suggesting that LCs may initiate tissue surveillance programs earlier during development.

Similar to the distinct expansion dynamics observed for both cell types, we found that DETCs underwent a gradual, tissue-specific maturation in the developing epidermis, whereas LCs progressed through a stepwise differentiation process, engaging distinct genetic programs. Early-stage DETCs exhibited high expression of cell cycle–associated and migratory genes, as well as markers of IFN-γ–producing γδ T cell lineages, consistent with residual thymic imprinting and a cytotoxic bias. This is in line with previous studies showing that neonatal DETCs preferentially produce IFN-γ upon activation in the perinatal epidermis (*51*). During DETC maturation, they progressively up-regulate genes involved in cytoskeletal organization and tissue repair, along with increased expression of gene sets related to dendrite development, reflecting their adaptation to a homeostatic surveillance role. In addition to known maturation programs, we also identified genes with poorly characterized roles in DETCs, such as *Ramp3*, *Gem*, and *Mest*. *Ramp3* encodes a receptor activity–modifying protein that regulates calcitonin gene–related peptide receptor trafficking, which has been linked to neuropeptide signaling in T cells, where it promotes T helper 1 differentiation (*72*). *Gem* (guanosine 5′-triphosphate–binding protein expressed in mitogen-stimulated T cells) is induced upon T cell activation and influences cytoskeletal remodeling, chemotaxis, and actin dynamics (*73*, *74*). In contrast, *Mest*, an imprinted gene associated with adipogenesis and mesenchymal development, has no established role in immune cells but may regulate epigenetic or metabolic programs (*75*, *76*). The enrichment of these genes in mature DETCs suggests potential contributions to niche adaptation or immunometabolic homeostasis, although their precise functions in γδ T cells remain to be elucidated.

In contrast to DETCs, LCs displayed distinct gene programs across development, indicating a stepwise differentiation process characterized by successive transcriptional states. First, our analyses demonstrated that LCs at E18.5 and P1 share many characteristics with activated macrophages and immature macrophage progenitors. Early LC progenitors are derived from fetal monocytes, migrate to the dermis at E16.5, and differentiate into macrophage progenitors within the tissue before entering the epidermis and developing into LCs (*20*, *23*). We found that LC progenitors transiently express core microglial signature genes and gene programs during the perinatal period, which disappear shortly thereafter. Recent studies have reported microglia-like progenitors in human fetal skin, testis, and heart (*53*, *68*). These microglia-like progenitors display signatures reminiscent of the microglial programs we observed in mouse LCs during the perinatal window. This suggests a specific imprinting of recruited progenitors by epidermal niche factors such as IL-34 or TGF-β1, analogous to imprinting mechanisms in the developing central nervous system. However, despite the continuous presence of these niche factors throughout postnatal development, the microglia-like signature in LCs vanishes shortly after birth (*77*, *78*). The stepwise developmental diversion of pre- and perinatally seeded skin immune cells away from their fetal programs is in line with that of dermal macrophages that mostly lose CX3CR1 expression with enduring tissue residency (*79*). The transient nature of this microglial signature may indicate that LC progenitors interact with neurons during early epidermal colonization, for example, by engaging with outgrowing sensory neurons in the dermis or using them as migratory tracks to access the epidermis (*68*). This is supported by previous reports of sciatic nerve–associated macrophages exhibiting microglia-like gene signatures despite eventually acquiring a niche-specific identity (*80*). Thus, dermal sensory neurons, which harbor sensory neuron-associated macrophages, may also serve as an anatomical hub for LC progenitors before their migration into the epidermis (*79*, *81*). Our data further indicate that core programs associated with LC maturation are established from P7 onward, as evidenced by the up-regulation of known LC marker genes such as *Cd207*, *Epcam*, and MHC-II–related genes. This supports the idea of early tissue imprinting that culminates around the time of weaning (P21), which appears to represent a developmental checkpoint for LC maturation. Subsequently, the acquisition of tissue-specific functions, such as cell-cell junction formation, reinforcement of epidermal barrier integrity, and immune surveillance, is further associated with the completion of this maturation process.

We did not directly assess interactions with keratinocytes, which produce key cytokines and mediate cell-cell contacts critical for epidermal immune cell development. IL-34, for example, is primarily produced by keratinocytes during both development and steady state and supports LC differentiation (*26*, *27*), whereas TGF-β1 can be derived from keratinocytes or produced via autocrine loops within LCs (*78*). Skint1, essential for the selection and maintenance of epidermal DETCs, is also predominantly expressed by keratinocytes (*30*, *52*). Thus, keratinocyte-derived signals may substantially contribute to the maturation of both cell types and warrant future investigation, ideally at single-cell resolution during fetal and postnatal development. Potential interactions between DETCs and LCs, allowing for coordinated postnatal maturation, have not yet been described. On the basis of our scRNA-seq data and ligand-receptor inference analyses, we identified DETC-derived signals, namely, *Il13*, *Csf2*, and *Tgfb1*, as candidate regulators of LC development and maturation. While TGF-β1 is well known to be essential for LC differentiation, DETCs have not previously been recognized as a source of this cytokine during epidermal development. IL-13 has been implicated in the regulation of dermal dendritic cell activation during steady state, but its potential role in LC maturation remains unexplored (*82*). Similarly, CSF2 (GM-CSF) is required for in vitro LC differentiation, yet its function in postnatal LC maturation in vivo is poorly defined (*83*, *84*). Thus, future studies are needed to elucidate whether DETC-derived CSF2, IL-13, or TGF-β1 contribute functionally to LC maturation under physiological conditions.

In contrast, functional testing in *Tcrd*^−/−^ mice, which lack γδ T cells, revealed no defects in LC maturation, as evidenced by an unchanged transcriptomic profile of LCs in the absence of γδDETCs in the adult epidermis. This observation may be explained by the compensatory presence of αβ T cells (αβDETCs) in *Tcrd^−/−^* mice, which likely preserve essential signaling cues required for LC differentiation. Supporting this, our cell-cell communication analysis showed that ligand-receptor interactions between αβDETCs and LCs closely mirrored those observed between γδDETCs and LCs, with no differences in interaction strength. Consistent with this, receptor expression and downstream signaling pathways in LCs, including IL-13, CSF2, and TGF-β signaling, remained largely unchanged in *Tcrd*^−/−^ mice. Nevertheless, αβDETCs are known to decline from the epidermis after ∼3 months of age (*85*), raising the possibility that long-term LC homeostasis could be impaired in aging *Tcrd^−/−^* mice. Future studies in aged *Tcrd^−/−^* mice or *Cd3e^−/−^* mice, which lack all T cells, are needed to clarify the extent to which DETC-LC interactions are dispensable for both development and maintenance. Our cell-cell interaction analysis identified LC-derived CCL4 as a potential signal toward DETCs. However, the predicted receptors on DETCs were CCR1 and CCR8, neither of which are known to directly bind CCL4 (*86*). This suggests that LC-to-DETC communication may be limited or indirect. Further investigation, such as LC depletion studies during postnatal development, will be necessary to determine whether LCs have any functional role in DETC maturation.

Our results further highlight that the maturation of the entire epidermal immune compartment is not affected by either the absence or increased complexity of microbiota during postnatal development. In other organs, the maturation of tissue-resident macrophages has been shown to depend on microbial cues and microbiota-derived metabolites (*81*, *87*). By contrast, other barrier tissues, such as the oral mucosa, exhibit marked microbiota-dependent differences in immune cell composition, ontogeny, and turnover (*35*). Whether commensal metabolites are excluded from the epidermal niche by the perinatally established basal membrane, or whether such metabolites are accessible but redundant for epidermal immune development, remains unresolved. Notably, DETCs and LCs have been implicated in tolerance induction to skin commensals, and LCs have been reported to scavenge microbiota and antigens from the skin surface by extending dendrites through the tightly connected keratinocyte layers (*12*, *88*, *89*). Despite these established functions, our data demonstrate that their own maturation is likely governed by tissue-intrinsic programs, with microbiota-derived signals appearing dispensable for this process. Because our study focused on the steady-state differentiation dynamics and interactions of DETCs and LCs, we cannot exclude the possibility that their interactomes during perinatal development contribute to the establishment of tissue immunity and commensal tolerance. Future studies are needed to investigate how the absence of DETCs or LCs affects immune responses to pathogens, tolerance to skin commensals, and the induction of self-tolerance during early life.

Invariant Vγ5^+^Vδ1^+^ DETCs dominate the murine epidermis but have no direct counterpart in humans, and human LCs differ in ontogeny and long-term maintenance (*8*, *67*, *90*). Similarly epidermal layering and developmental specification are different between mouse and human. Whereas the mouse epidermis specifies during late gestational stages shortly before birth, the human epidermis already starts specifying in much earlier fetal stages (*1*, *91*). These anatomical and developmental differences have often limited direct translational interpretation of murine epidermal immune studies. However, our cross-species analysis reveals that the core transcriptional maturation programs and intercellular signaling networks governing epidermal γδ T cell-LC interactions are conserved between mouse and human epidermis. In both species, γδ T cells transition from proliferative fetal states toward tissue repair programs, while LCs progress from microglia-like complement-enriched progenitor states toward MHC-II expressing antigen-presenting cells. The attribution of a microglia-like signature in LC progenitors is in line with recent studies highlighting microglia-like progenitors in fetal human skin (*53*, *68*). Moreover, conserved ligand-receptor modules, including IL-13, CSF2, TGF-β, integrin-mediated adhesion, TNF-family, and Notch signaling, structure bidirectional communication across developmental stages. These findings indicate that epidermal immune maturation follows evolutionarily conserved gene expression programs and signaling architectures in mouse and human skin despite marked species-specific differences in cellular composition and ontogeny. This conservation supports the use of murine models to dissect mechanistic principles of epidermal immune maturation while acknowledging species-specific contextual differences.

In summary, our study delineates developmental programming of DETCs and LCs during early life in mouse epidermis. By defining their distinct differentiation trajectories independent from microbial colonization and revealing compensatory signaling within the epidermal niche, we establish a comprehensive framework for understanding postnatal epidermal immune maturation. Moreover, our cross-species analyses demonstrate that core maturation programs and γδ T cell–LC signaling networks are conserved between mouse and human epidermis despite differences in cellular composition and ontogeny. Together, these findings provide a conceptual foundation for dissecting the principles that govern epidermal immune homeostasis and early-life tissue education.

## MATERIALS AND METHODS

### Study design

This study aimed to define the developmental trajectories and interaction networks of DETCs and LCs during fetal and postnatal epidermal maturation. We combined whole-mount imaging and scRNA-seq to profile epidermal immune cells from mouse skin across developmental stages from late embryogenesis to adulthood. To test whether canonical γδDETCs are required for LC maturation, we analyzed epidermal immune cells from *Tcrd*^−/−^ mice lacking γδ T cells and compared them with wild-type (WT) controls. To assess the influence of microbial colonization, we profiled epidermal immune cells from GF, SPF, and wildlings. A minimum of three biological replicates (animals) was included per experimental condition. For scRNA-seq analyses, we first identified immune populations through unsupervised clustering and subsequently separated DETCs and LCs for dedicated downstream analyses. For cross-species comparison, we reanalyzed publicly available human prenatal and adult skin scRNA-seq datasets and separated epidermal γδ T cells and LCs using canonical markers. Detailed descriptions of the computational workflows and data processing steps are provided in the respective Materials and Methods sections. Biological replicates and sample sizes are indicated in the respective figure legends.

### Mice

*C57BL/6J* mice from Charles River were used as WT mice. All mice were housed in specific pathogen–free conditions and backcrossed on *C57BL/6J* genetic background, if not otherwise stated in the manuscript. Animals were kept in a 12-hour/12-hour dark-light cycle, and water and food were provided ad libitum. All animal experiments were approved by local administration (X22/03A, X26/02B, and G23/017; Regierungspräsidium Freiburg) and were performed in accordance with the respective national and institutional regulations. Transgenic mouse lines including *Tcrd^−/−^* (*B6.129P2-Tcrdtm1Mom/J*) and *Cx3cr1^GFP^* [*B6.129P2(Cg)-Cx3cr1tm1Litt/J*] were used in this study (*63*, *92*). Wildlings (*C57BL/6NTac*) were created by embryo transfer of laboratory mice into pseudopregnant wild mice (*65*)*.* Germfree animals were obtained from the Clean Mouse Facility (Bern, Switzerland). These mice were born and raised in a completely sterile milieu in pressurized HEPA-filtered plastic film isolators (*93*).

### Timed mating

Timed mating was used to analyze embryos at defined developmental stages. The matings were started in the evening and separated after vaginal plug check on the following morning. Embryonic development was estimated considering the day of vaginal plug formation as E0.5 and staged by developmental criteria (*94*). Embryos were dissected at E17.5 and E18.5. Postnatal time points P0, P1, P2, P4, P7, P14, P21, P30, P60, and P90 and P100 were dissected referring to the day of birth.

### Dissection of peri- and postnatal epidermis

Pregnant mice were euthanized with CO_2_, followed by cervical dislocation. Embryos at E17.5 and E18.5, as well as neonates from P0 to P7, were decapitated, and ventral skin was dissected. For P4 and P7, hair was removed from abdominal skin using depilatory cream and mechanical detachment after 5 min. Skin samples were washed in ice-cold 1× phosphate-buffered saline (PBS). The skin was digested for 45 min at 37°C in enzyme mix containing Dispase II (2.4 mg/ml; Sigma-Aldrich) and 3% fetal bovine serum (FBS; Thermo Fisher Scientific) in 1× PBS. Afterward, the epidermis was carefully separated from the dermis using fine forceps.

### Dissection of adult epidermis

Mice at the age of P14 onward were euthanized with CO_2_, followed by cervical dislocation. Skin tissue from mice at P14 and older was obtained from ears. Hair was removed from ears with depilatory cream and mechanical detachment after 5 min. Skin samples were washed in ice-cold 1× PBS (Sigma-Aldrich). Ventral and dorsal sheets of the ears were separated. The skin was digested for 45 min at 37°C in enzyme mix containing Dispase II (2.4 mg/ml; Sigma-Aldrich) and 3% FBS in PBS. Afterward, epidermis was carefully separated from the dermis using fine forceps.

### Immunofluorescence imaging of whole-mount epidermis

Neonatal ventral epidermis was fixed for 2 hours, and ear epidermis was fixed for 20 min in 4% paraformaldehyde (Sigma-Aldrich) at 4°C. Epidermal samples were washed three times with 1× PBS and blocked for 2 hours at 4°C with 1 ml of blocking buffer containing 0.5% bovine serum albumin (BSA, Carl ROTH), 5% normal goat serum (7BioScience), and 0.3% Triton X-100 (Sigma-Aldrich) in 1× PBS. Epidermis was then incubated overnight at 4°C with primary antibody mix containing anti-Langerin (clone eBioRMUL.2, rat anti-mouse, eBioscience; 1:100), anti-EpCAM (clone G8.8, rat anti-mouse, Thermo Fisher Scientific; 1:300), anti-F4/80 (clone EPR2645-166, chicken anti-mouse, Abcam; 1:300), anti-CD3e (clone SP162, rabbit anti-mouse, Abcam; 1:300) and anti-Ki67 (clone SP6, chicken anti-mouse, Abcam and clone SolA15, rat anti-mouse, Thermo Fisher Scientific; 1:500) in blocking buffer containing 0.5% BSA and 0.3% Triton X-100 in 1× PBS. After rinsing three times with blocking buffer, the secondary antibody mix containing donkey anti-rat Alexa Fluor 488 (Thermo Fisher Scientific; 1:300), goat anti-chicken Alexa Fluor 488 (Thermo Fisher Scientific; 1:300), goat anti-rabbit Alexa Fluor 568 (Thermo Fisher Scientific; 1:300), donkey anti-rabbit Alexa Fluor 647 (Thermo Fisher Scientific; 1:300), goat anti-rat Alexa Fluor 647 (Thermo Fisher Scientific; 1:300), and 4′,6-diamidino-2-phenylindole (Thermo Fisher Scientific, 1:5000) was added and incubated for 2 hours at room temperature. Sheets were washed again three times with blocking buffer and subsequently mounted using Mowiol mounting medium (Thermo Fisher Scientific). LC and DETC numbers during development were quantified by confocal imaging with a SP8 X with white-light laser (Leica) using a 20× objective. Representative close-up images of LC and DETC development were taken using a 63× objective. Quantification of Ki67^+^ cells was performed using the Zeiss LSM 710 confocal microscope using a 20× objective and Z-stacks of 0.2 to 1 μm. We analyzed Z layers separately to identify Ki67^+^ DETCs and LCs and distinguish them from proliferating basal stem cells in the epidermis. For imaging of DETCs and LCs in wildling samples, a Leica Thunder Imager with a 20× objective was used. Representative images of epidermal sheets from wildlings and SPF animals were taken with the Zeiss LSM 710 Confocal using a 20× objective. Postacquisition editing and quantification of all representative figures were done with the Fiji platform (*95*).

### scRNA-seq of the epidermal tissue

Isolated epidermis was incubated for 20 min at 37°C in enzyme mix containing collagenase D (1 mg/ml; Sigma-Aldrich), deoxyribonuclease (0.2 mg/ml; Sigma-Aldrich), and 3% FBS in PBS. Epidermis was mechanically homogenized through a 100-μm cell strainer into FACS buffer containing 0.5% BSA and 2 mM EDTA (Sigma-Aldrich) in PBS. Tubes for the collected cells were precoated with 10% BSA for 2 hours. Cells were centrifuged at 320*g* for 7 min at 4°C and resuspended in Fc receptor blocking antibody anti-CD16/32 (clone 2.4G2, BD Biosciences; 1:200) for 20 min on ice to prevent nonspecific antibody binding. Afterward, cells were incubated with anti-Gr1 (clone RB6-8C5, BD Biosciences; 1:200) and anti-CD19 (clone 6D5, BioLegend; 1:200) to exclude granulocytes and B cells and an anti-CD45 (clone 30-F11, eBioscience; 1:200) antibody to gate on DETCs and LCs. Fixable Viability Dye eFluor 780 (eBioscience; 1:1000) was used to exclude dead cells. In addition, 1 μl of hashing antibodies was added per sample. Hashing antibodies were obtained as purified and already oligo-conjugated in TotalSeq-C (5′ chemistry) format from BioLegend. Cells were stained for 20 min and washed. CD45^+^ immune cells were sorted using a BD FACSAria III, a BD FACSAria Fusion, or a Beckman Coulter MoFlo Astrios EQ in the Lighthouse Core Facility, University of Freiburg. Sorted cells were processed through the 10x Genomics single-cell 5′ v2 workflow according to the manufacturer’s instructions. Libraries were pooled to desired quantities to obtain appropriate sequencing depths as recommended by 10x Genomics and sequenced on a NovaSeq6000 sequencer (Illumina Inc.).

### Quantification of transcript abundance and downstream scRNA-seq data analysis

Gene expression and hashtag abundance were quantified using the count command from cellranger-6.0.0 and cellranger-7.2.0 using the prebuilt CellRanger mouse mm10 references from 2020. The scRNA-seq data were analyzed using Python (v3.12.4) and the packages scanpy (v1.10.4) and muon (v0.1.6) (*96*, *97*). The hashtags were demultiplexed using hashsolo as included in scanpy, and batch effects were removed using Harmony (*98*, *99*). Cells containing less than 500 counts or 200 genes or more than 10% mitochondrial counts were removed as low-quality cells. Genes that appeared in less than three cells were also removed. Ribosomal and mitochondrial genes, as well as predicted genes with the *Gm*-identifier, were excluded from the analysis after the quality control. The counts were normalized and set to a target sum of 10,000, followed by log1p transformation and scaling with a cutoff at 10. To determine the top 2000 highly variable genes, the flavor “seurat_v3” was used in the highly_variable_gene function. Dimensionality reduction was performed using uniform manifold approximation and projection (UMAP) while making sure that the neighborhood graph was calculated on the Harmony-corrected principal components analysis embedding. Clustering was performed using the leiden algorithm, and clusters were annotated on the basis of marker genes found after calculating DEGs. For downstream analysis of DETCs only canonical Vγ5^+^Vδ1^+^ DETCs (expressing *Tcrg-V5* and *Trdv4*) were kept.

### Trajectory analysis of scRNA-seq data

To include temporal information in the trajectorial analysis, a temporal problem from Moscot was built and passed to CellRank to build a real-time kernel (*49*, *100*–*102*). In addition, a connectivity kernel was built using the neighborhood graph. These were combined into a single kernel. Then, a generalized probabilistic canonical correlation analysis estimator was created using the combined kernel, and initial and terminal states were predicted. Because multiple terminal states were predicted, the trajectory leading to the terminal state closest to our last time point was picked as the differentiation trajectory of interest. The initial state was used to determine the root cell for diffusion pseudotime calculation based on a PAGA graph at cluster resolution. DPT was then used as the trajectory to smooth the expression of genes. Smoothed gene profiles were used for clustering by building an AnnData object and clustering using the Leiden algorithm as implemented in CellRank and scanpy.

### Cell-cell communication inference from scRNA-seq data

Cell-cell communication was inferred using the packages LIANA and Tensor-cell2cell (*58*, *103*). For this purpose, the resource “mouseconsensus” from LIANA was used in combination with the methods logfc, geometric_mean, singlecellsignalr, connectome, cellphonedb, natmi, and cellchat. Subsequent analysis was performed using a consensus score from all these methods as implemented in LIANA. These methods were used to calculate a consensus score for all replicates separately, and the results were used to build a 4D tensor using Tensor-cell2cell. This tensor was then decomposed into several factors, which was picked according to package instructions.

### Human scRNA-seq data analysis

Publicly available human prenatal and adult skin scRNA-seq datasets (ArrayExpress accessions E-MTAB-8142 and E-MTAB-7407) were downloaded and processed using the same analysis pipeline as described for the murine datasets unless otherwise stated (*68*, *69*). Cells annotated as immune populations were first subsetted and subjected to standard quality control filtering. From these immune compartments, we further identified and subsetted LCs and γδ T cell–like populations based on canonical marker expression. Because spatial annotation distinguishing epidermal and dermal compartments was not available for fetal samples, cell populations were selected on the basis of transcriptional identity rather than tissue annotation. After filtering and subsetting, datasets were integrated across donors using Harmony for batch correction at the donor level. Downstream analyses, including dimensionality reduction, clustering, gene expression analysis, pathway scoring, and ligand-receptor interaction inference using LIANA and Tensor-cell2cell, were performed as described above for the murine datasets.

### Statistical analysis

All statistical analyses were performed using Python (version 3.12.4). When normality assumptions were not met, we used two-sided nonparametric Mann-Whitney *U* tests for comparisons between two groups unless otherwise specified. For multiple testing scenarios, *P* values were adjusted using the Benjamini-Hochberg procedure to control the false discovery rate. For scRNA-seq analyses, differential gene expression between clusters was computed using a Wilcoxon rank sum test adjusted by Bonferroni correction. Differential gene expression in pseudobulk samples was calculated using PyDESeq2, accounting for batch effects where applicable. Gene set activity scores were computed using AUCell. Ligand-receptor interaction analyses were performed using LIANA and Tensor-cell2cell. Sample sizes and number of biological replicates are indicated in the figure legends. Differences were considered statistically significant at adjusted *P* < 0.05 unless otherwise stated. Box plots show the distribution across biological replicates. Dots represent individual biological replicates (independent mice). The center line indicates the median, the box boundaries represent the 25th and 75th percentiles [interquartile range (IQR)], and whiskers extend to 1.5 × IQR. Numerical source data underlying the main and supplementary figure plots are provided in data file S19.
