# Supplementary material for: Distinct postnatal trajectories of mouse dendritic epidermal T cells and Langerhans cells independent of microbiota
Source: Sci Adv. 2026 Aug 14;12(33):eaec9180. doi: 10.1126/sciadv.aec9180 (PMC13475618; doi:10.1126/sciadv.aec9180)
Supplement: Supplementary file 1 [file sciadv.aec9180_sm.pdf]

Science Advances
AAAS

Supplementary Materials for

Distinct postnatal trajectories of mouse dendritic epidermal T cells and Langerhans cells independent of microbiota

David Obwegs *et al.*

Corresponding author: Katrin Kierdorf, katrin.kierdorf@uniklinik-freiburg.de; Sagar, sagar@uniklinik-freiburg.de

Sci. Adv. **12**, eaec9180 (2026)
DOI: 10.1126/sciadv.aec9180

The PDF file includes:

Figs. S1 to S7
Legends for data files S1 to S19

Other Supplementary Material for this manuscript includes the following:

Data files S1 to S19

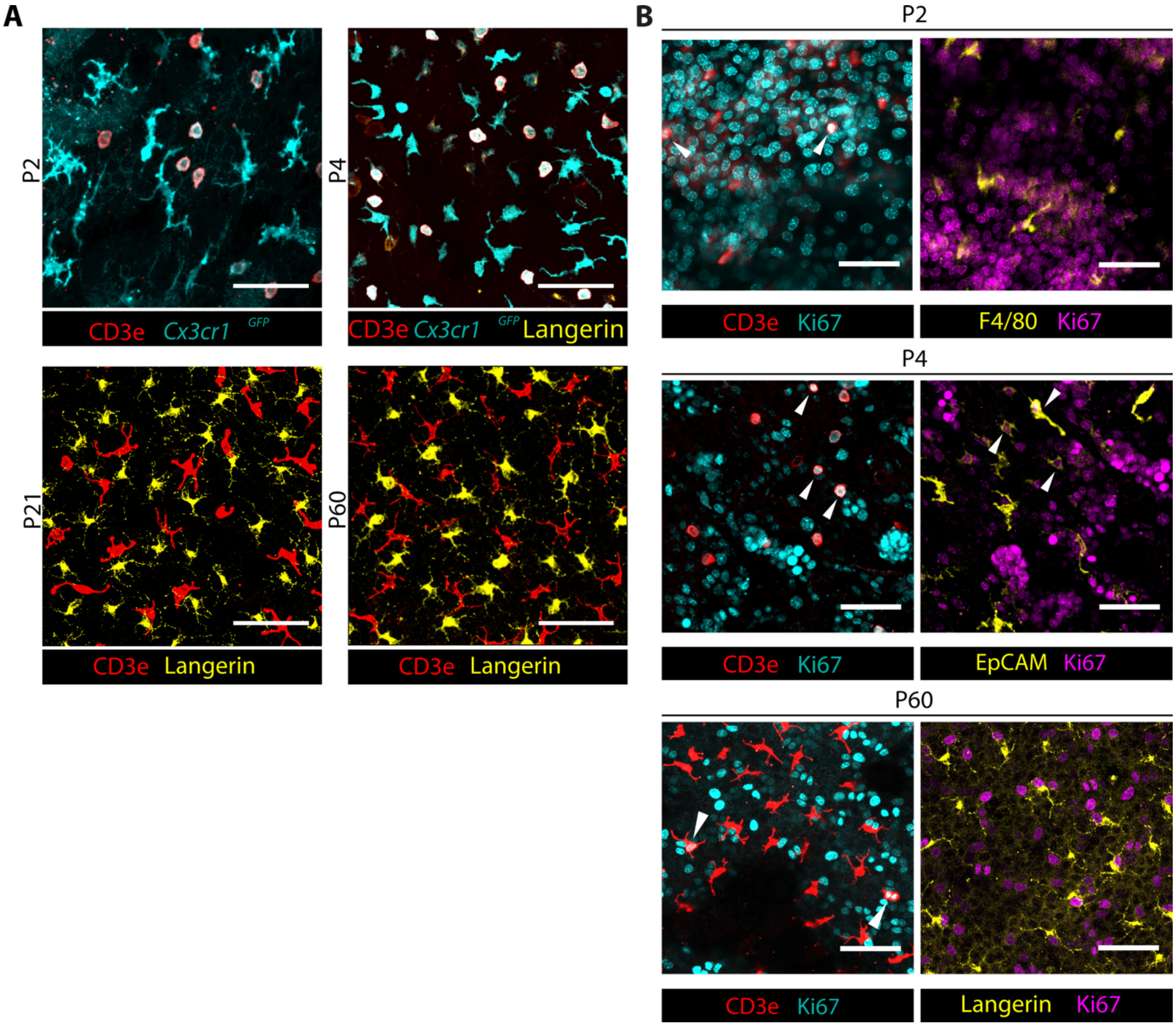

Fig. S1. Epidermal networks of DETCs and LCs expand postnatally. (A) Representative whole mount confocal images of epidermal LCs and DETCs at analyzed time points. CD3e is shown in red, *Cx3cr1*^{GFP} is shown in cyan, and Langerin is shown in yellow. Maximum projection of the confocal z-stack is shown. Scale bar = 50 µm. (**B)** Representative whole mount confocal images of epidermal Ki67^{+} LCs and DETCs at analyzed time points. CD3e is shown in red, Ki67 is shown in cyan or magenta, and F4/80, EpCAM or Langerin are shown in yellow. Arrowheads indicate proliferating DETC or LC. Selected z layers are shown for illustration purposes. Scale bar = 50 µm.

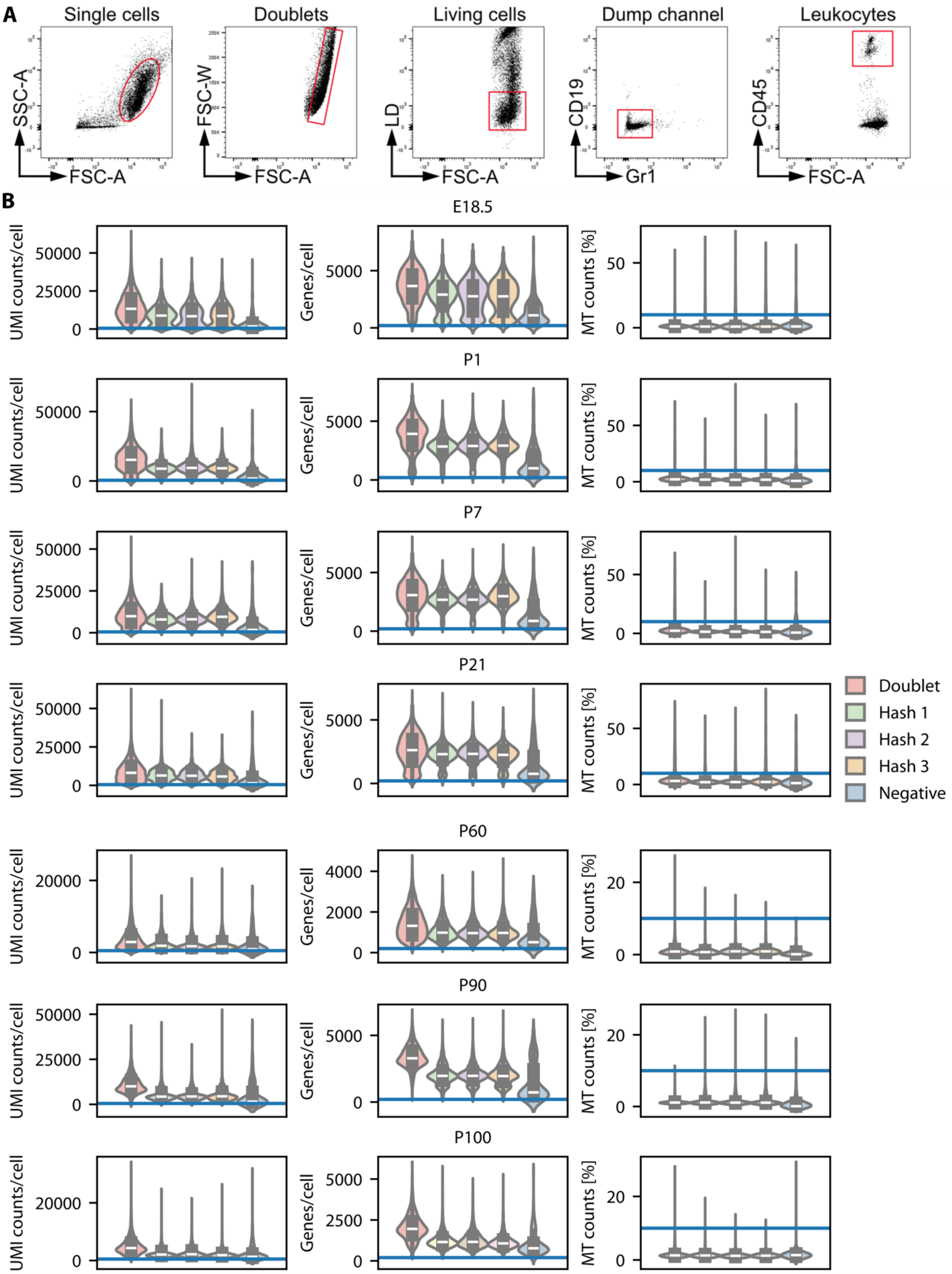

Fig. S2. Epidermal immune cell isolation and quality control of scRNA-seq data. (A) Representative flow cytometry plots showing the gating strategy used for single cell sorting of

immune cells from epidermal tissues. (**B)** Violin plots depicting the number of UMI counts (left), number of genes (middle), and the percentage of mitochondrial genes (right) per cell after demultiplexing separated by assignment to either a hashing antibody, doublet or negative. Color is indicated in legend. Blue horizontal lines indicate the thresholds used for quality filtering, and cells falling outside these thresholds were excluded from further analyses.

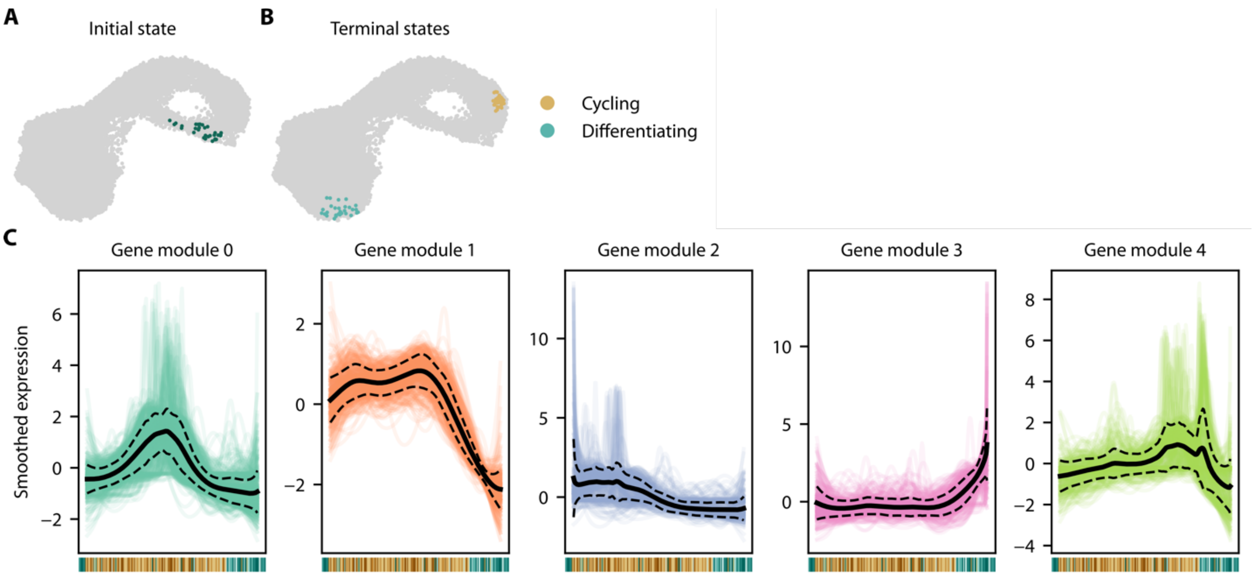

Fig. S3. Trajectory analysis of DETCs across development. (**A**) UMAP plot showing the initial state (green) as predicted by the package CellRank. (**B)** UMAP plot showing the terminal state (cycling (orange) and differentiated (cyan)) as predicted by the package CellRank. (**C)** Gene modules as identified by Leiden clustering of smoothed gene expression of top 2000 highly variable genes along pseudotime. Continuous line represents the mean of all genes in the module and dashed lines represent the mean ± standard deviation. Colors represent the respective modules as shown in Fig. 3, H and I.

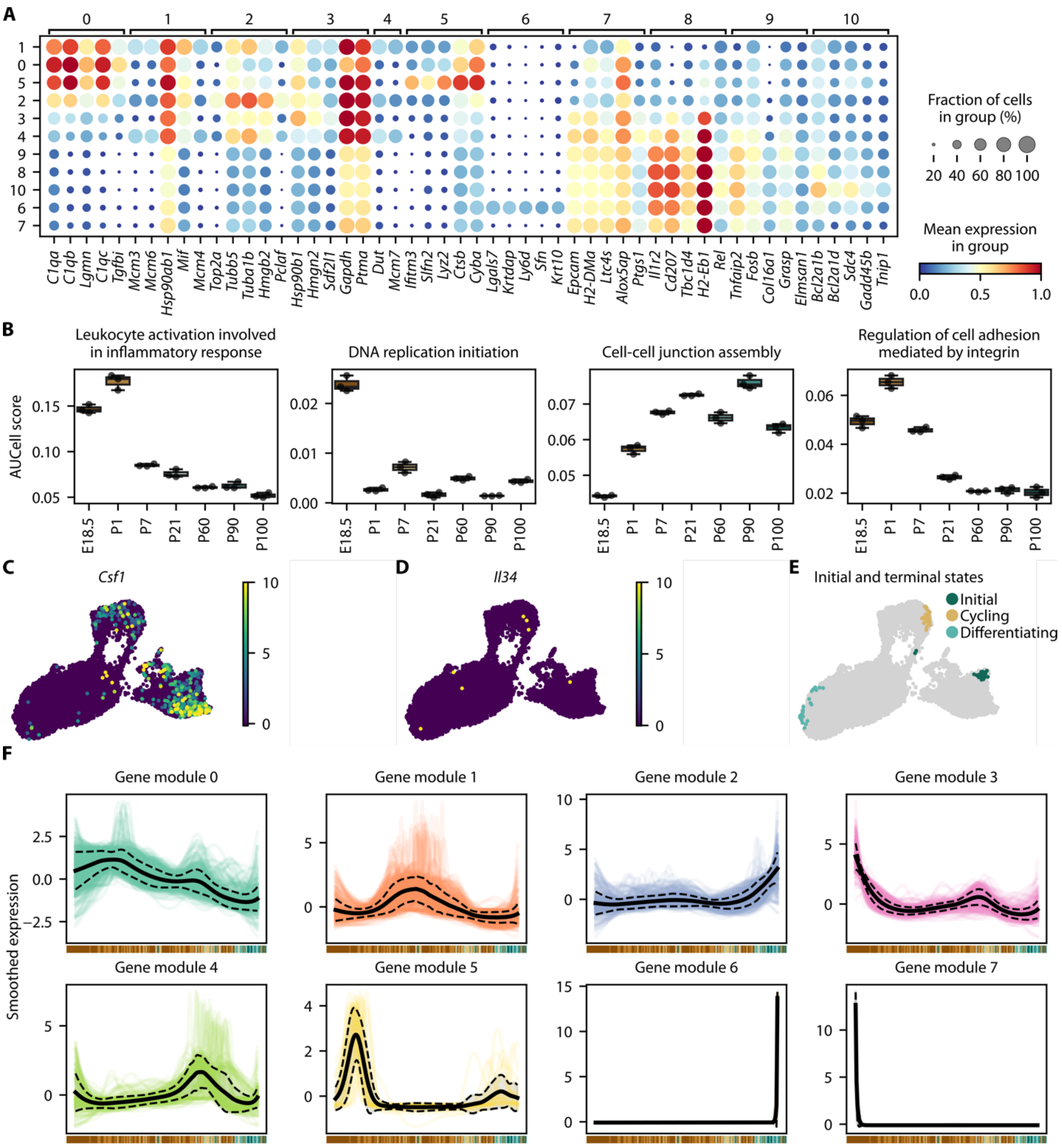

Fig. S4. LC employ distinct gene programs during their postnatal maturation. (**A**) Dot plot of top 5 DEGs per cluster. Color represents scaled mean expression of the gene in the respective cluster and dot size represents the fraction of cells expressing the gene. Cluster number is indicated in legend. (**B)** Box plot showing mean AUCell scores per replicate of selected GO terms across the different time points. Dots represent the mean value of each biological replicate (independent mouse). The center line indicates the median, the box boundaries represent the 25th and 75th percentiles (interquartile range, IQR), and whiskers extend to 1.5 × IQR. (**C**) Expression of *Csf1* on UMAP embedding based on gene expression profiling. Scale reflects the relative gene expression. (**D)** Expression of *Il34* on UMAP embedding based on gene expression profiling. Scale reflects the relative gene expression. (**E**) UMAP plot depicting the initial (green), terminal (cycling

(orange) and differentiating (cyan) states as predicted by CellRank indicated by color. **(F)** Gene modules as identified by Leiden clustering of smoothed gene expression of top 2000 highly variable genes along DPT. The continuous line represents the mean of all genes in the module and dashed lines show the mean ± standard deviation. Colors represent the respective modules as shown in Fig. 4, H and I.

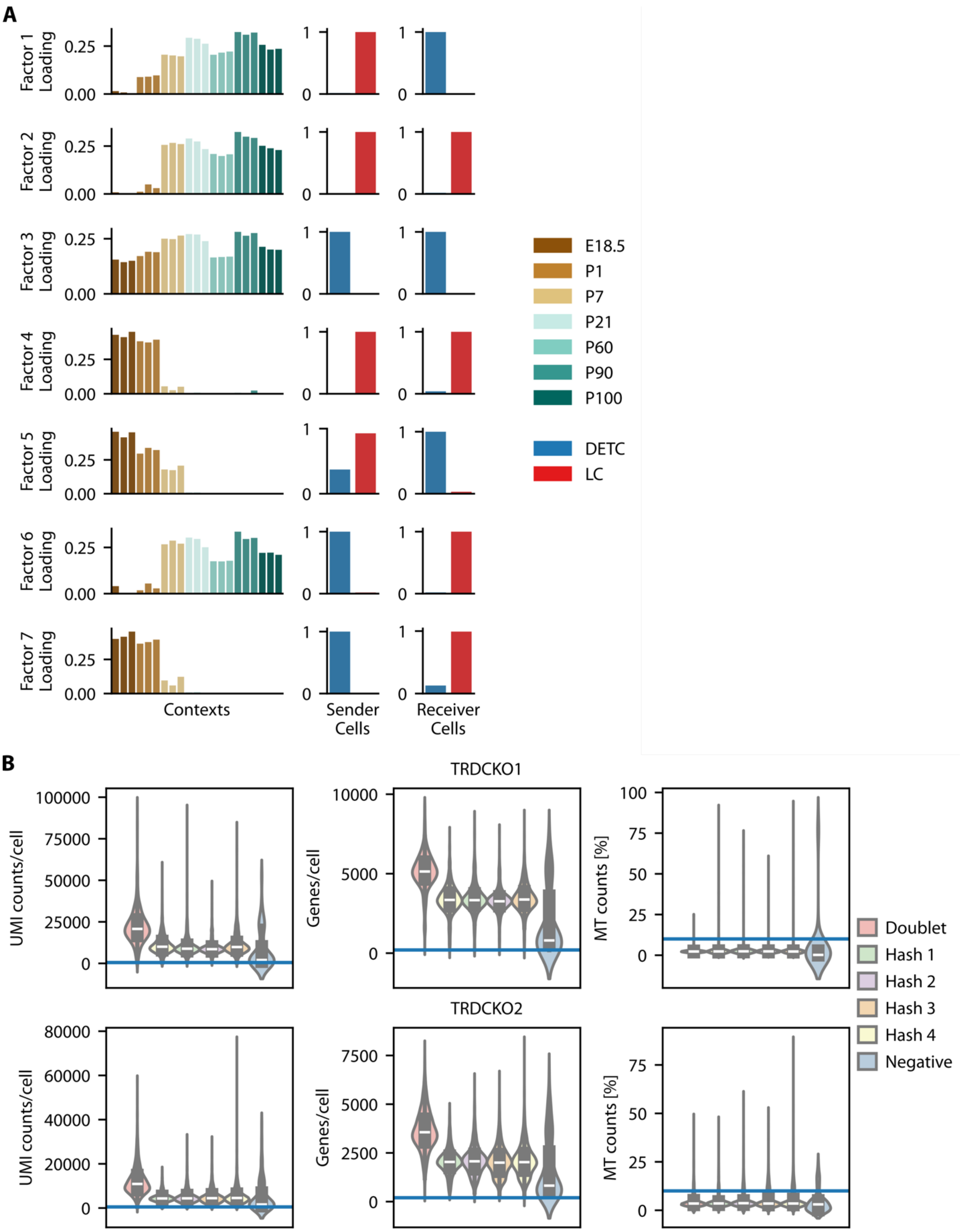

Fig. S5. LCs and DETCs display a defined and concise interactome across development. (A). Graphs depicting results of running Tensor-cell2cell frameworks on our dataset containing different time points. Each row represents a factor displaying paracrine or autocrine signaling

between DETC (blue) and LC (red), and each column a tensor dimension, wherein each bar plot represents an element of that dimension (time point, a sender cell or a receiver cell). Factor loadings (y-axis) are displayed for each element of a given dimension. Colors of individual time points are depicted in legend. (**B)** Violin plots depicting the number of UMI counts (left), number of genes (middle), and the percentage of mitochondrial genes (right) per cell after demultiplexing for *Tcrd*$^{-/-}$ mice separated by assignment to either a hashing antibody, doublet or negative. Color is indicated in legend. Blue horizontal lines indicate the thresholds used for quality filtering, and cells falling outside these thresholds were excluded from further analyses. Blue horizontal lines indicate the thresholds used for quality filtering, and cells falling outside these thresholds were excluded from further analyses.

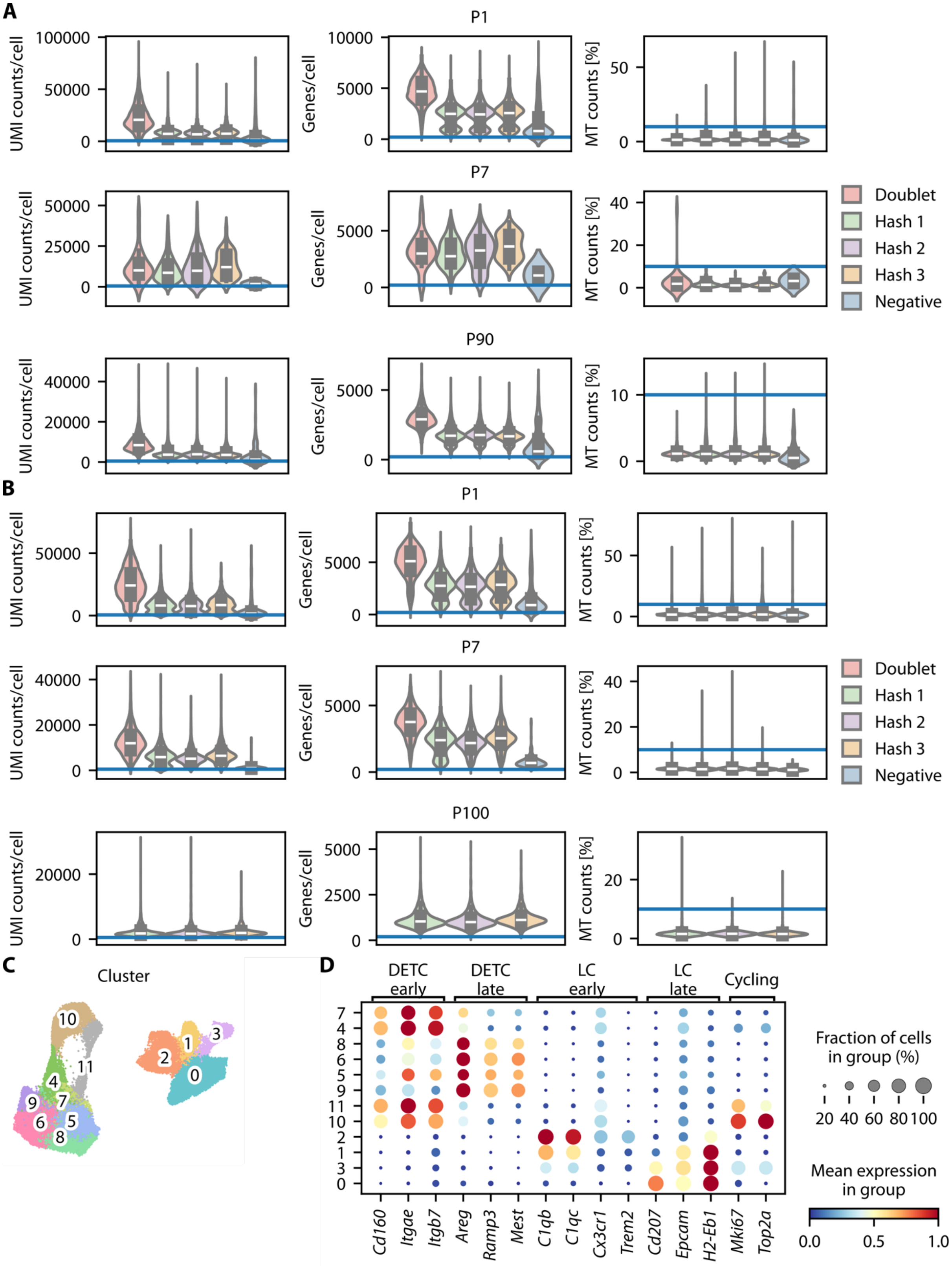

Fig. S6. Microbial colonization of the skin does not affect DETC and LC maturation. (A) Violin plots depicting the number of UMI counts (left), number of genes (middle), and the percentage of mitochondrial genes (right) per cell after demultiplexing for germfree mice separated

by assignment to either a hashing antibody, doublet or negative. Color is indicated in legend. (**B)** Violin plots showing the number of UMI counts (left), number of genes (middle), and the percentage of mitochondrial genes (right) per cell after demultiplexing for wildlings separated by assignment to either a hashing antibody, doublet or negative. Color is indicated in legend. Blue horizontal lines indicate the thresholds used for quality filtering, and cells falling outside these thresholds were excluded from further analyses. (**C)** UMAP plot showing 12 identified cell clusters of GF mice, SPF mice and wildlings (N = 80556 cells). Each color represents one cell cluster. **(D)** Dot plot showing expression of selected genes in the 12 different clusters for early and late DETCs, LCs and cycling cells. Color represents the scaled mean expression of the gene in the respective cluster and dot size represents the fraction of cells in the cluster expressing the gene.

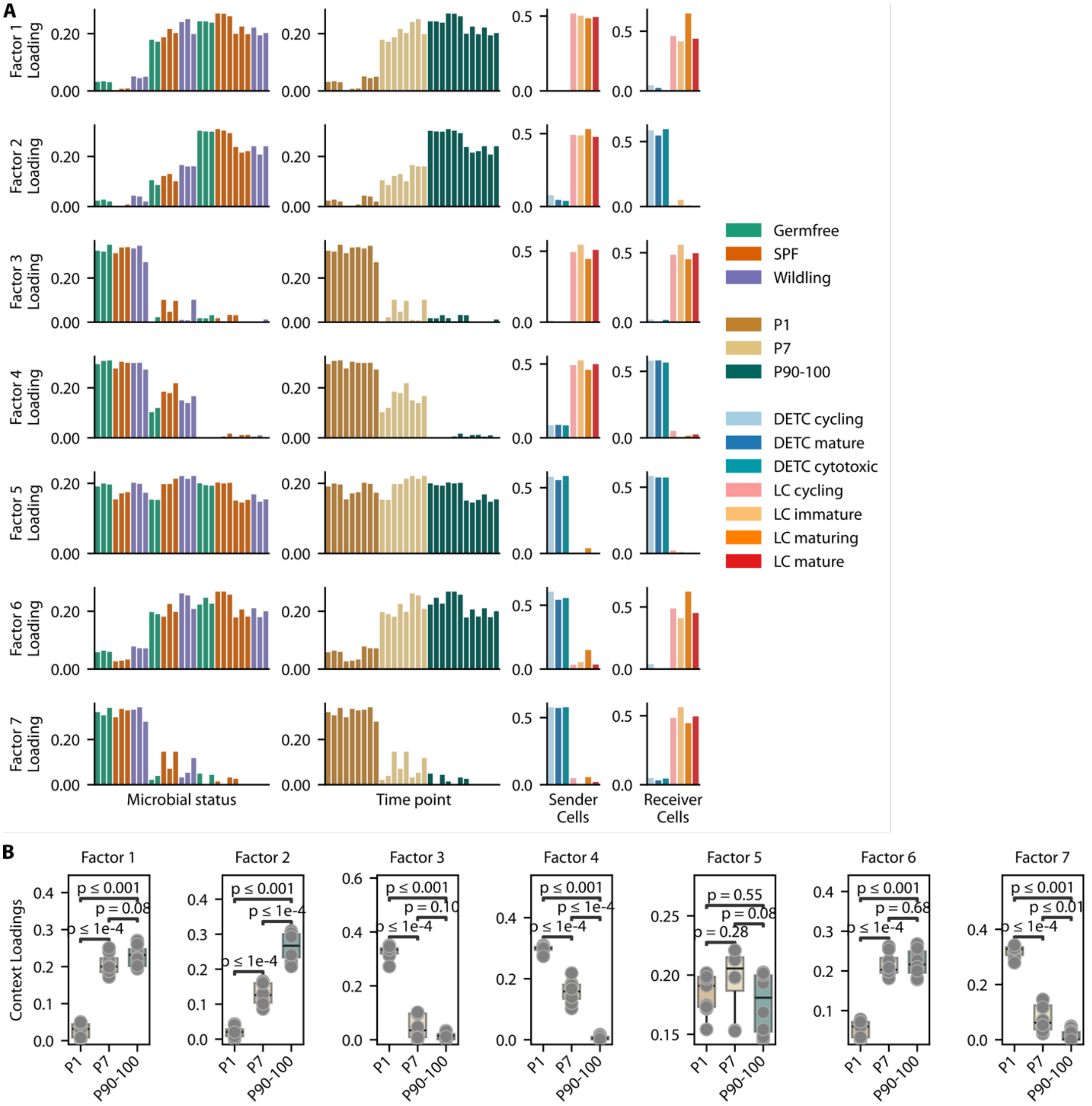

Fig. S7. Developmental time point rather than microbial status defines DETC and LC maturation. (**A**) Graphs depicting results of running Tensor-cell2cell frameworks on our dataset containing different microbial statuses, time points and cell states (cycling, immature, maturing, mature and cytotoxic). Each row represents a factor, and each column a tensor dimension, wherein each bar plot represents an element of that dimension (microbial status, time point, a sender cell or a receiver cell). Factor loadings (y-axis) are displayed for each element of a given dimension. Color is indicated in legend. (**B**) Box plots of the context loadings for all seven factors recovered by factor decomposition of a tensor built using microbial status and time points as contexts. Dots represent the mean value of each biological replicate (independent mouse). The center line indicates the median, the box boundaries represent the 25th and 75th percentiles (interquartile range, IQR), and whiskers extend to 1.5 × IQR. Statistical testing was performed using Mann-Whitney U rank test with Benjamini-Hochberg correction for multiple testing.

Separate files

Data file S1. Table containing results from differential expression analysis for clusters seen in Fig. 2B. The table reports statistics for all genes tested after pre-filtering steps applied prior to differential expression analysis. Genes with an adjusted *P* value < 0.05 were considered significant.

Data file S2. Table containing the gene sets used to score cells for their cell cycle status in Fig. 2F.

Data file S3. Table containing results from differential expression analysis for clusters seen in Fig. 3A. The table reports statistics for all genes tested after pre-filtering steps applied prior to differential expression analysis. Genes with an adjusted *P* value < 0.05 were considered significant.

Data file S4. Table containing the gene sets used to score cells in Fig. 3E.

Data file S5. Table containing the gene modules determined using CellRank and depicted in Fig. 3H.

Data file S6. Table containing results from differential expression analysis for clusters seen in Fig. 4A. The table reports statistics for all genes tested after pre-filtering steps applied prior to differential expression analysis. Genes with an adjusted *P* value < 0.05 were considered significant.

Data file S7. Table containing the gene modules determined using CellRank and depicted in Fig. 4H.

Data file S8. Table containing the loadings for all factors derived from tensor decomposition using LIANA and Tensor-cell2cell as seen in Fig. 5A.

Data file S9. Table containing results from differential expression analysis for clusters seen in Fig. 5C. The table reports statistics for all genes tested after pre-filtering steps applied prior to differential expression analysis. Genes with an adjusted *P* value < 0.05 were considered significant.

Data file S10. Table containing results from differential expression analysis between genotypes for LCs as seen Fig. 5, I and J, determined using PyDESeq2 on counts pseudo-bulked by replicate and including batch as a confounding variable are included. The table reports statistics for all genes tested after pre-filtering steps applied prior to differential expression analysis. Genes with an adjusted *P* value < 0.05 were considered significant.

Data file S11. Table containing the gene sets used to score cells in Fig. 5K.

Data file S12. Table containing the loadings for all factors derived from tensor decomposition using LIANA and Tensor-cell2cell as seen in Fig. 6A.

Data file S13. Table containing results from differential expression analysis between genotypes for DETCs determined using PyDESeq2 on counts pseudo-bulked by replicate and including batch as a confounding variable are included. The table reports statistics for all genes tested after pre-filtering steps applied prior to differential expression analysis. Genes with an adjusted *P* value < 0.05 were considered significant.

Data file S14. Table containing results from differential expression analysis for clusters seen in fig. S6C. The table reports statistics for all genes tested after pre-filtering steps applied prior to differential expression analysis. Genes with an adjusted *P* value < 0.05 were considered significant.

Data file S15. Table containing the loadings for all factors derived from tensor decomposition using LIANA and Tensor-cell2cell as seen in fig. S7A.

Data file S16. Table containing results from differential expression analysis for clusters seen in Fig. 8B. The table reports statistics for all genes tested after pre-filtering steps applied prior to differential expression analysis. Genes with an adjusted *P* value < 0.05 were considered significant.

Data file S17. Table containing the gene sets used to score cells in Fig. 8, G and H.

Data file S18. Table containing the loadings for all factors derived from tensor decomposition using LIANA and Tensor-cell2cell as seen in Fig. 9A.

Data file S19. Numerical source data for main and supplementary figures.
